# The tumor suppressor HNRNPK induces p53-dependent nucleolar stress to drive ribosomopathies

**DOI:** 10.1172/JCI183697

**Published:** 2025-05-08

**Authors:** Pedro Aguilar-Garrido, María Velasco-Estévez, Miguel Ángel Navarro-Aguadero, Álvaro Otero-Sobrino, Marta Ibáñez-Navarro, Miguel Ángel Marugal, María Hernández-Sánchez, Prerna Malaney, Ashley Rodriguez, Oscar Benitez, Xiaroui Zhang, Marisa J.L. Aitken, Alejandra Ortiz-Ruiz, Diego Megías, Manuel Pérez, Gadea Mata, Jesús Gomez, Miguel Lafarga, Orlando Domínguez, Osvaldo Graña-Castro, Eduardo Caleiras, Pilar Ximénez-Embun, Marta Isasa, Paloma Jimena de Andres, Sandra Rodríguez-Perales, Raúl Torres-Ruiz, Enrique Revilla, Rosa María García-Martín, Daniel Azorín, Josune Zubicaray, Julián Sevilla, Oleksandra Sirozh, Vanesa Lafarga, Joaquín Martínez-López, Sean M. Post, Miguel Gallardo

**Affiliations:** 1 Department of Hematology, Hospital Universitario 12 de Octubre, Instituto de Investigación Sanitaria Hospital 12 de Octubre (imas12), Madrid, Spain.; 2 H12O-CNIO Haematological Malignancies Clinical Research Unit, CNIO, Madrid, Spain.; 3 Department of Biochemistry and Molecular Biology, Universidad Complutense de Madrid, Madrid, Spain.; 4 Department of Leukemia, MD Anderson Cancer Center, Houston, Texas, USA.; 5 Department of Biochemistry and Cell Biology, Dartmouth Cancer Center, Geisel School of Medicine at Dartmouth, Lebanon, New Hampshire, USA.; 6 Confocal Microscopy Unit, CNIO, Madrid, Spain.; 7 Department of Anatomy and Cell Biology and Centro de Investigación Biomédica en Red sobre Enfermedades Neurodegenerativas (CIBERNED), University of Cantabria-IDIVAL, Santander, Spain.; 8 Genomics Unit and; 9 Bioinformatics Unit, CNIO, Madrid, Spain.; 10 Institute of Applied Molecular Medicine, Department of Basic Medical Sciences, School of Medicine, San Pablo-CEU University, CEU Universities, Boadilla del Monte, Madrid, Spain.; 11 Histopathology Unit and; 12 Proteomics Unit, CNIO, Madrid, Spain.; 13 Department of Medicine and Surgery, School of Veterinary Medicine, Universidad Complutense de Madrid, Madrid, Spain.; 14 Molecular Cytogenetics Unit, CNIO, Madrid, Spain.; 15 Division of Hematopoietic Innovative Therapies, Biomedical Innovation Unit; Centro de Investigaciones Energéticas Medioambientales y Tecnológicas and Centro de Investigación Biomédica en Red de Enfermedades Raras (CIEMAT/CIBERER); and Advanced Therapies Unit, Instituto de Investigación Sanitaria Fundación Jiménez Díaz, Madrid, Spain.; 16 Department of Pathology, Hospital 12 de Octubre, Madrid, Spain.; 17 Department of Pathology and; 18 Onco-Hematology Department, Hospital Infantil Universitario Niño Jesús, Fundación para la Investigación Biomédica del Hospital Infantil Universitario Niño Jesús, Madrid, Spain.; 19 Genomic Instability Group, CNIO, Madrid, Spain.; 20 Department of Medicine, Universidad Complutense de Madrid, Madrid, Spain.

**Keywords:** Aging, Hematology, Stem cells, Cellular senescence, Hematopoietic stem cells, Mouse models

## Abstract

The nucleolus is a membraneless organelle and an excellent stress sensor. Any changes in its architecture or composition lead to nucleolar stress, resulting in cell cycle arrest and interruption of ribosomal activity, critical factors in aging and cancer. In this study, we identified and described the pivotal role of the RNA-binding protein HNRNPK in ribosome and nucleolar dynamics. We developed an in vitro model of endogenous HNRNPK overexpression and an in vivo mouse model of ubiquitous HNRNPK overexpression. These models showed disruptions in translation as the HNRNPK overexpression caused alterations in the nucleolar structure, resulting in p53-dependent nucleolar stress, cell cycle arrest, senescence, and bone marrow failure phenotype, similar to what is observed in patients with ribosomopathies. Together, our findings identify HNRNPK as a master regulator of ribosome biogenesis and nucleolar homeostasis through p53, providing what we believe to be a new perspective on the orchestration of nucleolar integrity, ribosome function and cellular senescence.

## Abstract 2

Hnrnpk is a novel driver of nucleolar stress, ribosomopathies and aging-like phenotype.

## Graphical abstract

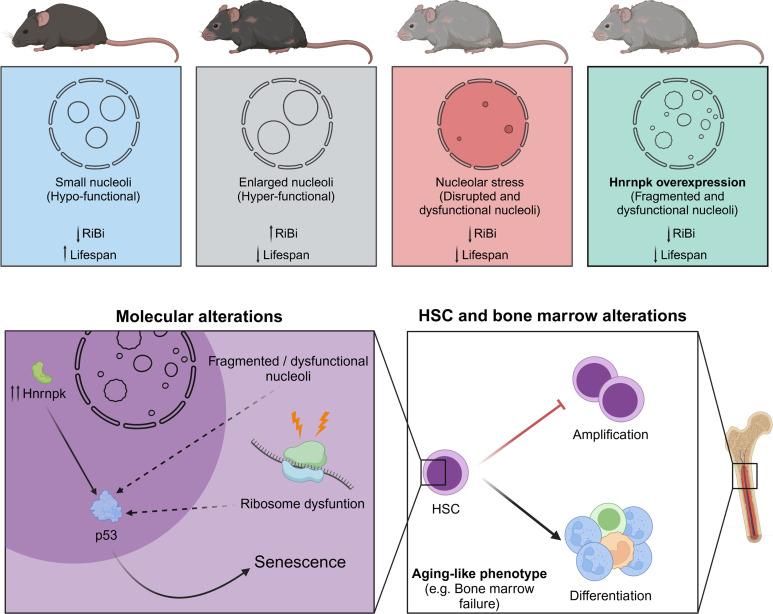

## Introduction

The nucleolus is the biological structure where ribosomal subunits are generated. Structured in 3 regions, the fibrillar centers, dense fibrillar component, and granular component, it tightly regulates the functional assembly of rRNA and RNA-binding proteins (RBPs) (1, 2). Although the primary function of the nucleolus is being the hub for ribosome biogenesis (RiBi), it is also implicated in other cellular processes such as telomere regulation, genome maintenance, or cell cycle progression. Aberrancies in the nucleolar structure lead to a halt in its functions (1, 2). Likewise, the nucleolus acts as a stress sensor, and any defect in RiBi, translation, or DNA damage, among others, leads to nucleolar stress and disruption of its structure (1, 2).

The rRNAs account for approximately 80% of the total RNA generated in the cell, carrying an enormous energetic cost. As a reduction in cell energetic consumption is linked to longer lifespan (3), the nucleolus plays a main role in lifespan regulation; a reduction in nucleolar components or smaller nucleoli and activity is associated with prolonged lifespan (4), while enlarged functional nucleoli is associated with cancer and aging (1). Nucleolus and ribosome activity are tightly linked, and, as such, nucleolar stress and ribosome stress are intimately related, although the sources and consequences can differ. The molecule p53 is central in the response to multiple cellular stress insults, causing cell cycle arrest (5). There are several nucleolar and ribosomal proteins involved in the stabilization of p53 (6). Any dysregulation in the ribosome or nucleolus, leading to stress, will trigger an increase in p53 levels (7), while p53 inactivation rescues ribosome impairment consequences in multiple model systems (8). However, although p53 is clearly involved in the link between ribosomal and nucleolar stress, it does not explain all cases; there is a lack of knowledge about what other molecular players can regulate this relationship.

The RBPs are critical players in nucleolar activities. Among them, we find HNRNPK. This RBP is involved in RiBi, interacting with mRNA transcripts and noncoding RNAs (9), forming ribonucleoprotein complexes and binding nascent (10–12) transcripts for nuclear processing and subsequent export to ribosomes (13). It is also involved in nucleolar function (14) and interacts with nucleolar components such as nucleolin (4, 15). Therefore, HNRNPK is associated with both nucleolar and ribosome function and is potentially a key participant in their crosstalk.

Previous work has demonstrated the involvement of HNRPK in cancer and its dual nature, behaving as both an oncogene and a tumor suppressor by directly regulating key molecules like p53 and c-MYC (16, 17). Thus, HNRNPK has an important role in cell fate, governing the path toward neoplasia or senescence and aging. Although HNRNPK overexpression in B cells promotes tumorigenesis, its haploinsufficiency in the hematopoietic cell (HSC) context drives leukemias. Therefore, it is uncertain what the effect of HNRNPK overexpression on the HSC would be.

Nucleolar stress and ribosomal stress have been observed as hallmarks of multiple pathologies, including cancer, aging, neurological diseases, and ribosomopathies (5, 18). The latter are characterized by ribosome dysregulation and abnormalities in different tissues, like some but not all bone marrow failures, such as those occurring in myelodysplastic syndrome or Diamond-Blackfan anemia (19). HSCs are especially sensitive to these aberrancies, their amplification and proliferation are affected, which leads to exhaustion and final differentiation (20), a common phenotype of aged HSCs (19). Here, we showed that the overexpression of HNRNPK causes an impairment in ribosome function and biogenesis, nucleolar abnormalities, and senescence both in vitro and in vivo, consistent with a p53-dependent nucleolar stress response. In our mouse model, HNRNPK overexpression decreases mouse lifespan owing to bone marrow failure in a ribosomopathy-like phenotype. Therefore, with this study, we provide evidence of the role of HNRNPK in the nucleoli-ribosome axis, triggering a ribosomopathy-like phenotype in vivo.

## Results

### HNRNPK overexpression disrupts translation.

**Figure 1:**
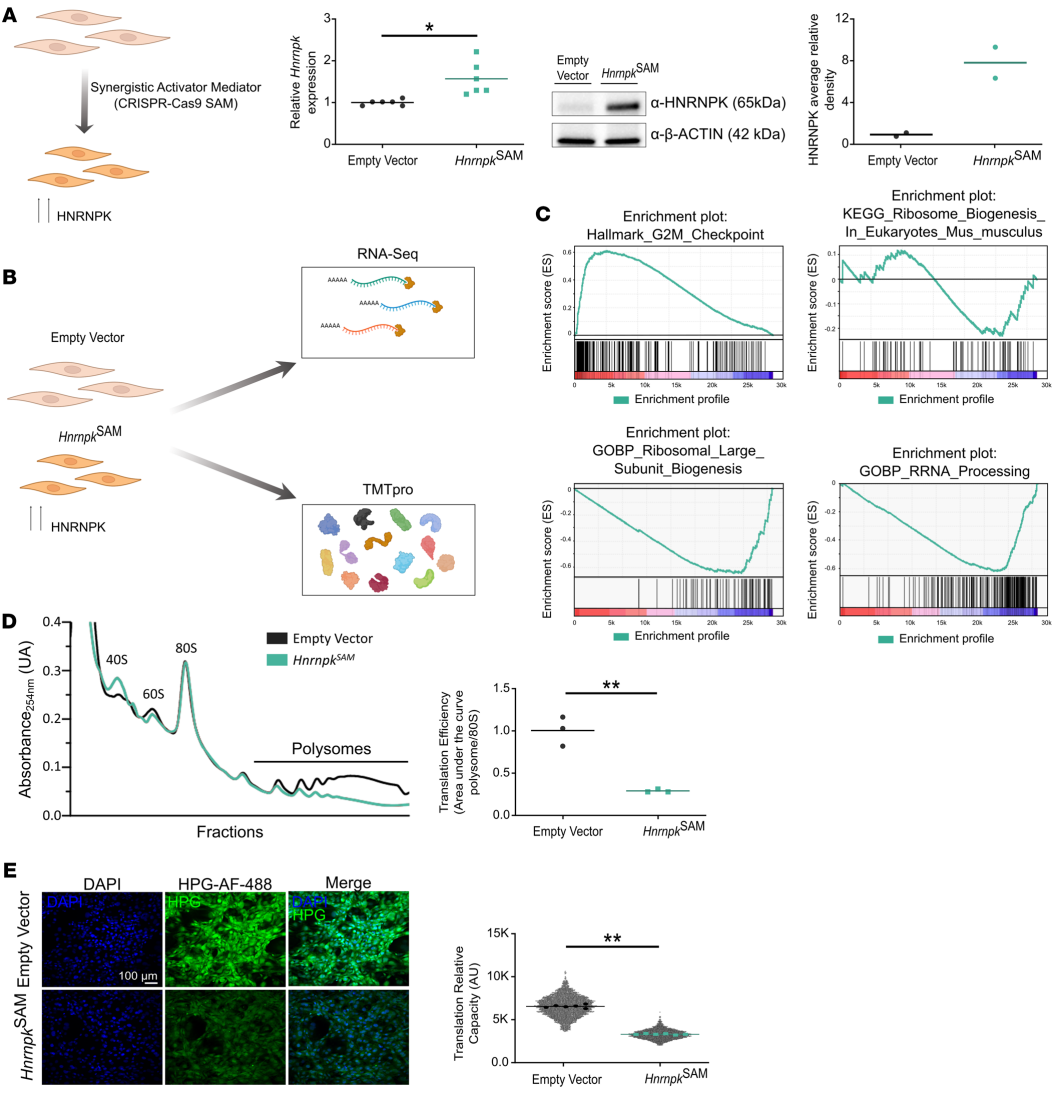
HNRNPK-overexpressing MEF generation and phenotyping showed a decrease in translation efficiency and capacity. (**A**) HNRNPK-overexpressing CRISPR_CAS9 SAM MEF (*Hnrnpk*^SAM^) model development diagram, *Hnrnpk* qRT-PCR box plot in *Hnrnpk^SAM^* MEFs (*P* = 0.0381), and HNRNPK Western blot membrane and densitometry box plot. (**B**) RNA-Seq and TMTpro diagram analysis of *Hnrnpk^SAM^* (*n* = 6) vs. empty vector MEFs (*n* = 3 and *n* = 4, respectively). (**C**) Top: Gene set enrichment analysis (GSEA) of RNA-Seq results showing enrichment plots of the top significantly regulated pathways (MsigDB): HALLMARK_G2M_CHECKPOINT (FDR *q* < 10^–4^, normalized enrichment score [NES] = 1.99) and KEGG_RIBOSOME_BIOGENESIS_IN_EUKARYOTES_MUS_MUSCULUS (FDR *q* < 0.05, NES = –1.89) in transcriptomes of *Hnrnpk*^SAM^ (*n* = 6) vs. empty vector MEFs (*n* = 3). Bottom: GSEA of TMTpro results showing enrichment plots of the top significantly regulated pathways (MsigDB) GOBP_RIBOSOMAL_LARGE_SUBUNIT_BIOGENESIS (FDR *q* < 10^–4^, NES = –25.05) and GOBP_RRNA_PROCESSING (FDR *q* < 10^–4^, NES = –28.94) in proteomes of *Hnrnpk*^SAM^ (*n* = 6) vs. empty vector MEFs (*n* = 4). A FDR *q* value of less than 0.05 was chosen as a cutoff for exploratory data analysis. (**D**) Left: Representative polysome profiles of *Hnrnpk^SAM^* and an empty vector sample. Right: *Hnrnpk^SAM^* (*n* = 3) vs. empty vector (*n* = 3) translation efficiency values (area under the curve of polysome fraction/area under the curve of 80S fraction) (*P* = 0.002). (**E**) Left: Representative images from the HPG assay. Right: Fluorescence intensity values (dot plot, > 1,000 cells per representative well; biological replicate analysis, *P* = 0.0022). All graphs are shown as the mean or median. Two-sided Student’s *t* test, *P < 0.05; **P < 0.01. All experiments comprised at least *n* = 3 biological replicates and/or *n* = 3 technical replicates.

To study the impact of HNRNPK overexpression on biological programs, we developed two models of mouse embryonic fibroblasts (MEFs) that constitutively overexpress HNRNPK: one generated using CRISPR/Cas9/synergistic activator mediator (SAM) technology (*Hnrnpk*^SAM^) (Figure 1A) and another generated by inserting the *Hnrnpk* gene in a *pCALL2* vector (*Hnrnpk*^Tg-Cre^). We confirmed *Hnrnpk* transcription and protein overexpression (Figure 1A and Supplemental Figures 1A and 5, A–D; supplemental material available online with this article; https://doi.org/10.1172/JCI183697DS1).

We performed RNA-Seq and multiplexed mass spectrometry–based proteomics (TMTpro) on the *Hnrnpk*^SAM^ MEFs (Figure 1B; Supplemental Figures 1, B and C; and Supplemental Tables 1, 2, 4, and 5). The most differentially expressed gene set, as determined by the Molecular Signatures Database (MSigDB; https://www.gsea-msigdb.org/gsea/msigdb) (21), was the G_2_/M checkpoint pathway, with upregulation of G_2_/M pathway genes in the HNRNPK-overexpressing model (RNA-Seq data, Figure 1C). Moreover, our HNRNPK-overexpressing models showed a significant enrichment of a set of downregulated genes and proteins involved in RiBi and rRNA processing (derived from MsigDB) (RNA-Seq and TMTpro data, Figure 1C).

To confirm the negative impact of HNRNPK on RiBi and function, we performed polysome quantification. We observed an overall decrease in translational efficiency (Figure 1D; Supplemental Figures 1D and 5, B–E), confirmed by a decrease in translational efficiency through a L-homopropargylglycine (HPG) assay (Figure 1E), pointing to a severe inhibition of global translation in HNRPK-overexpressing cells.

### HNRNPK overexpression drives nucleolar abnormalities with a nucleolar stress signature.

In our model, the combination of downregulated ribosome biosynthesis with an interruption of translation upon HNRNPK overexpression suggests the existence of a ribosomopathy. We hypothesized that the synthesis, processing, or assembly of ribosomal components could be dysregulated in the nucleolus.

**Figure 2:**
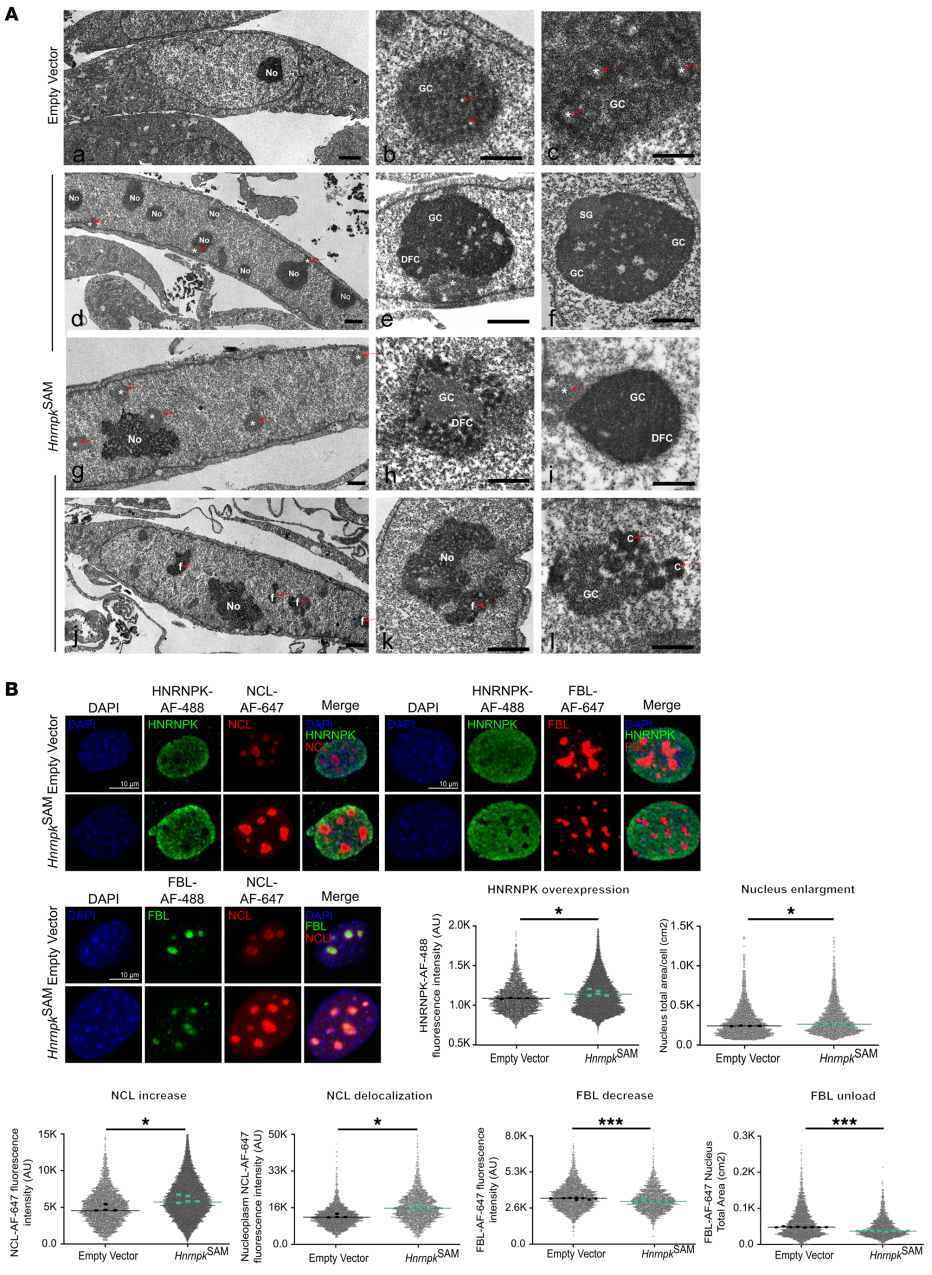
HNRNPK overexpression promotes nucleolar fragmentation and dysregulates nucleolar components. (**A**) Top row: Electron micrographs of control MEF (empty vector). Typical spindle-shaped MEF with a euchromatic nucleus and a prominent nucleolus (No) (empty vector, *n* = 2; *Hnrnpk*^SAM^, *n* = 8) (left). Representative example of a normal nucleolus with several fibrillar centers (*) (middle). Detail of a nucleolus showing several typical fibrillar centers surrounded by an annular shell of dense fibrillar component intermingled with the granular component (GC) (right). Second row left: Electron micrographs of MEFs overexpressing *Hnrnpk*^SAM^. Formation of multinucleolated MEFs. The nucleus shows multiple nucleoli and peripheral masses of heterochromatin (*) and nucleolar segregation and compaction of nucleolar components. SG, segregated nucleolar components. DFC, dense fibrillar component. Nucleolar fragments (f). Nucleolar caps (c). Scale bars: 500 nm (top right image); 1 μm (all other images). (**B**) Representative confocal microscopy images with NCL, HNRNPK, and DAPI and with FBL, HNRNPK and DAPI as well as representative confocal microscopy images of FBL, NCL, and DAPI staining (nuclei were stained with DAPI; biological replicates analysis, *P* = 0.020). HNRNPK expression was analyzed by Alexa Fluor 488 intensity (biological replicates analysis, *P* = 0.010); NCL expression and NCL nucleoplasm expression were accessed by dot plot analysis of Alexa Fluor 647 intensity (biological replicates analysis, *P* = 0.031); FBL expression was analyzed by dot plot analysis of Alexa Fluor 647 intensity (biological replicates analysis, *P* = 0.0002); and FBL unload was examined via Alexa Fluor 647 spot total area (biological replicates analysis, *P* = 0.0001); for all graphs, dot plot cell replicates, >1.000, *P < 0.05; ***P < 0.001. Scale bars: 10 μm. All graphs are shown as the mean or median.

By nucleolin immunolabeling (nucleolar marker) and electron microscopy analysis in our HNRNPK-overexpressing cells, we observed an increased number of nucleoli (up to 9 in a nuclear section) as well as smaller nucleoli (Figure 2). This observation was consistent with the increase in G_2_/M phase (Figure 1C), and the presence of polyploid plus aneuploid cells, which result in additional chromosomes carrying nucleolar organizer regions (22). Importantly, electron microscopy analysis also revealed the existence of nucleolar alterations in our HNRNPK-overexpressing model, including (a) segregation of components (dense fibrillar component, fibrillar centers) at the nucleolar periphery, (b) abnormal accumulations of the granular component, (c) lobulation and nucleolar fragmentation, and (d) formation of prominent masses of repressive heterochromatin (Figure 2A). Nucleolar abnormalities were confirmed by immunolabeling and determination of mRNA and protein levels of fibrillarin (FBL). We observed segregation of FBL in multiple foci throughout the nucleus and lower levels of expression (Figure 2, A and B and Supplemental Figures 2A and 5C). To further explore our hypothesis, we analyzed the expression pattern of nucleolin (NCL). This protein is a renowned stress sensor, and an increase in NCL expression has been correlated with nucleolar stress (23). We observed that NCL was overexpressed and partially translocated to the nucleoplasm (Figure 2B and Supplemental Figure 2, B and C), a delocalization commonly found to be p53 dependent, and considered to be one of the hallmarks of nucleolar stress (23–25). Interestingly, we corroborated the direct interaction between HNRNPK and NCL by IP, suggesting a direct regulation (Supplemental Figure 2D).

Finally, to confirm that HNRNPK overexpression triggers nucleolar stress hallmarks, we induced nucleolar stress in WT MEFs by treatment with actinomycin D. This transcriptional inhibitor mimicked the HNRNPK-overexpression signature: after nucleolar stress induction, HNRNPK was overexpressed, the nucleolar expression and nucleoplasmic delocalization signal of NCL were increased, and the fluorescent signal of FBL was reduced (Supplemental Figure 2E).

### HNRNPK overexpression dysregulates RiBi components.

To verify the effect of HNRNPK on RiBi and rRNA processing as well as the aberrancies shown in the nucleolus, we performed an analysis of the two ribosomal components: rRNAs and ribosomal proteins (RPLs and RPs).

**Figure 3:**
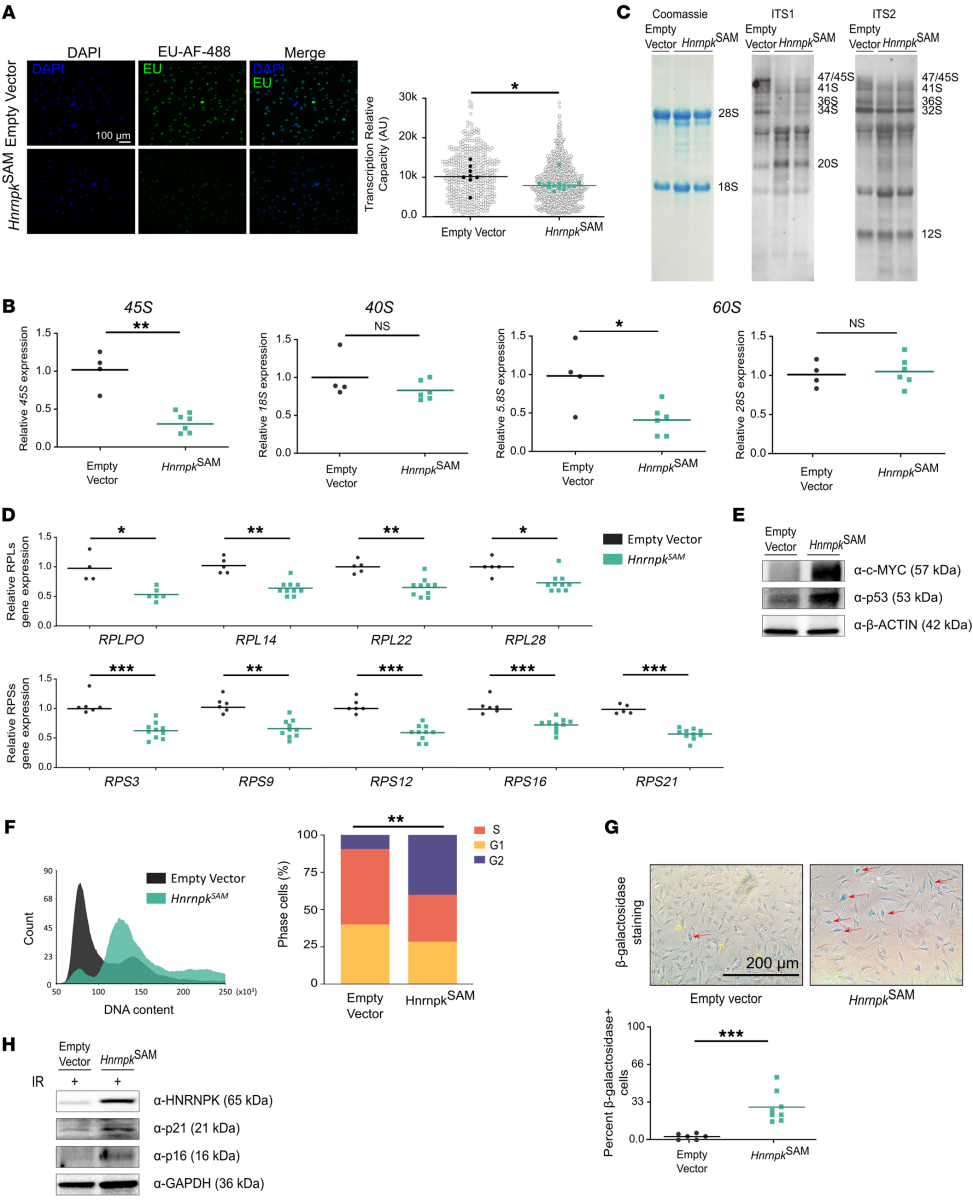
HNRNPK overexpression showed rRNA and ribosome protein reduction and drove cell cycle arrest and senescence. (**A**) Left: Representative images of EU assay. Right: Fluorescence intensity values (dot plot, >1.000 cells of representative well; biological replicates analysis: *P* = 0.013). Scale bar: 100 μm. (**B**) *Hnrnpk*^SAM^ (*n* = 6) vs. empty vector (*n* = 3) MEF qRT-PCR results, with dot plot showing pre-rRNA *45S* and mature rRNA transcripts *18S*, *28S*, and *5.8S* (*45S*, *P* = 0.0095; *5.8S*, *P* = 0.0397; *18S*, *P* = NS; *28S*, *P* = NS). (**C**) Northern blot membranes with ITS1 and ITS2 probes showing lower levels of 47S/45S rRNAs and aberrant levels of pre-RNA precursors such as 20S. (**D**) *Hnrnpk*^SAM^ (*n* ≥ 5) vs. empty vector (*n* ≥ 4) qRT-PCR results, with dot plot showing RPL (top) and RPS (bottom) gene expression (*Rpl22*, *P* = 0.0027; *Rpl14*, *P* = 0.0010; *Rpl28*, *P* = 0.0063; *Rps9*, *P* = 0.0020; *Rps21, P* = 0.0003; *Rps16, P* = 0.0007; *Rps12, P* = 0.0003; *Rps3, P* = 0.0007; *Rplpo, P* = 0.0095). (**E**) Western blot membrane of HNRNPK-overexpressing cells showing higher c-MYC and p53 expression. (**F**) Left: Representative cell cycle FCM histogram plots in *Hnrnpk*^SAM^ (*n* = 9) vs. empty vector (*n* = 9) MEFs. Right: Bar graph of percentage of G_1_, S and G_2_/M cells (*P* = 0.005) from FCM analysis. (**G**) Left: Bright-field microscope images of SA-β-galactosidase staining in *Hnrnpk*^SAM^ MEFs. Right: Dot plot of cells positive for SA-β-galactosidase staining in *Hnrnpk*^SAM^ (*n* = 8) vs. empty vector (*n* = 6) (*P* = 0.0007). Scale bar: 200 μm. (**H**) Western blot membrane of irradiated *Hnrnpk*^SAM^ cells showing an increase in the senescence markers p21 and p16. All graphs are shown as the median (**A**) or mean (**B**–**E**). Two-sided Student’s *t* test, with the exception of FCM cell cycle analysis (2-way ANOVA): *P < 0.05; **P < 0.01; ***P < 0.001. All experiments comprised at least *n* = 3 biological replicates and/or *n* = 3 technical replicates.

First, we performed an 5-ethynyl uridine (EU) in situ transcription assay. We observed a significant reduction in both nucleolar (pre-rRNA) and chromatin (pre-mRNA) transcription in *Hnrnpk*^SAM^ cells (Figure 3A). Moreover, the EU incorporation signal for nascent rRNA appeared within numerous small nucleoli with low fluorescence intensity, compared with larger nucleoli with high EU signal intensity found in the control, empty vector MEFs (Figure 3A). Since most of the nascent RNA within the cell is rRNA (80%), we analyzed the expression level of several rRNAs to confirm the previous EU data. Indeed, we observed lower levels of pre-rRNA 45S and large subunit rRNAs 5.8S (Figure 3B).

We performed Northern blot analysis to investigate the impact that HNRNPK has on rRNA transcription and processing. HNRNPK overexpression led to a reduction of murine 45S/47S pre-rRNA, alongside processing abnormalities demonstrated by expression differences in the rRNA precursors 20S and 34S (Figure 3C). In addition, we observed that HNRNPK is able to bind directly to rRNAs (Supplemental Figure 2D). These findings suggest that HNRNPK overexpression disrupts normal ribosomal biogenesis, leading to altered rRNA processing and aberrant rRNA precursor levels. Next, we corroborated a reduction in RPLs and RPs by qRT-PCR (Figure 3D).

### Nucleolar stress derived from HNRNPK overexpression promotes cell cycle arrest and senescence via p53.

Translation interruption, RiBi dysregulation, and nucleolar stress converge in the canonical stress response through p53, which was directly regulated by HNRNPK (5, 17, 23) (Figure 3E). Likewise, c-MYC, whose expression is positively regulated by HNRNPK, was observed to be overexpressed (Figure 3E). To zoom in on p53 regulation, we analyzed the cell cycle profile and apoptosis state in our cell models. We confirmed an increase in the G_2_/M phase (Figure 3F), as seen in RNA-Seq data (Figure 1C), and observed polyploid cells in *Hnrnpk*^SAM^ cells (Supplemental Figure 2F), consistent with the electron microscopy observations (Figure 2A). To investigate this further, we performed a karyotype analysis. We observed a gain in chromosome number and chromosomal abnormalities (Supplemental Figure 2F) in the HNRNPK-overexpressing model. We confirmed that this cell cycle arrest drove cells to senescence by SA-β-galactosidase (Figure 3G). Likewise, molecular partners of p53 that can drive cell cycle arrest were upregulated in our model (Figure 3H). Next, we evaluated the apoptosis levels by annexin V analysis, ratifying an increase in apoptotic cells (Supplemental Figure 2G) and cleaved caspase-3 (Supplemental Figure 2H) via p53 when HNRNPK was overexpressed.

As we observed chromosomal and DNA aberrancies such as polyploidy (Figure 2A and Supplemental Figure 2F), we corroborated that p53 overexpression was not due to genomic instability, DNA damage response, and/or replicative stress by the analysis of ɤ-H2AX (Supplemental Figure 2I).

Taken together, these results suggest that HNRNPK promotes nucleolar stress, which in this specific case is p53 dependent and causes p53-mediated cell cycle arrest, driving cells to senescence and apoptosis.

### The HNRNPK-overexpressing phenotype is p53 dependent.

**Figure 4:**
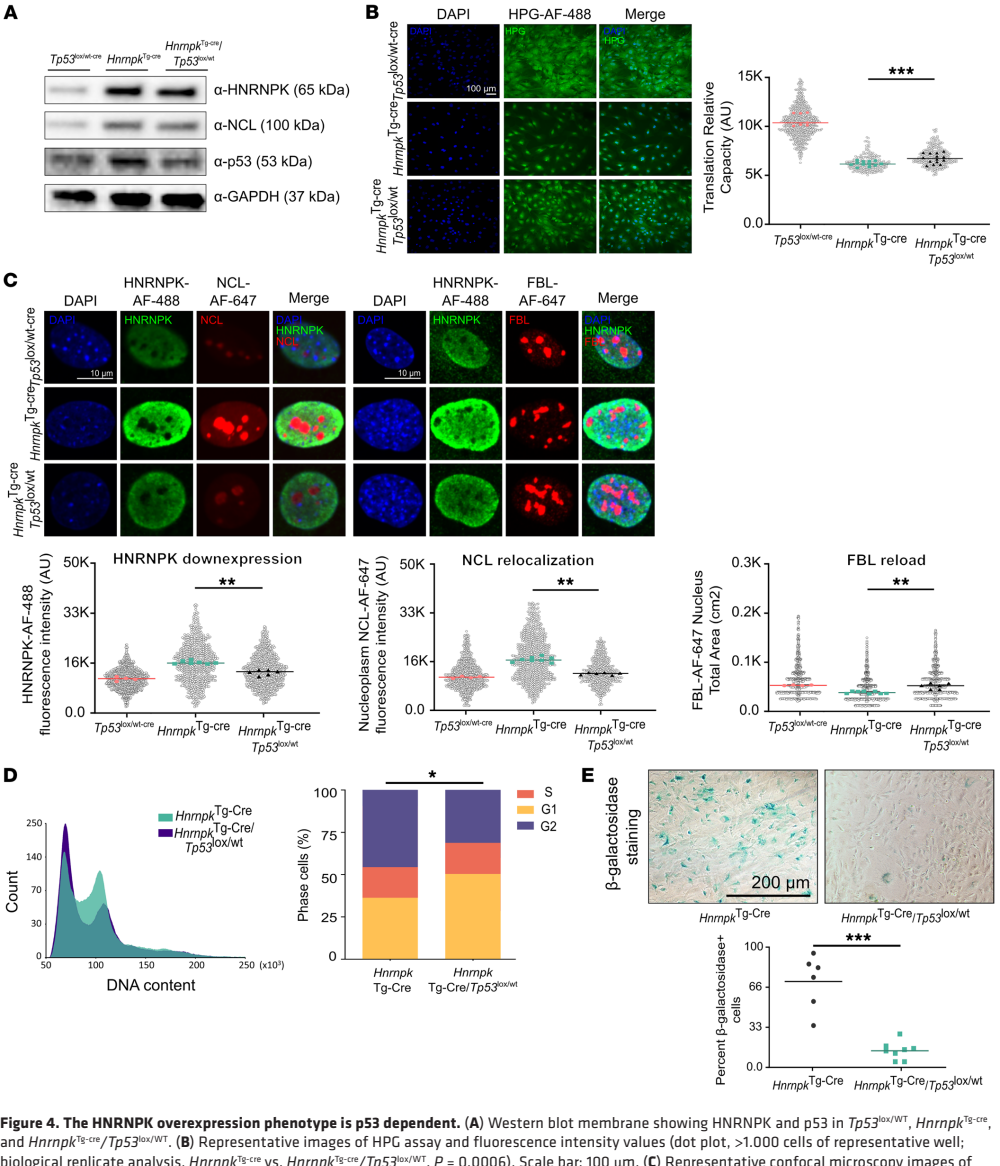
The HNRNPK overexpression phenotype is p53 dependent. (**A**) Western blot membrane showing HNRNPK and p53 in *Tp53*^lox/WT^, *Hnrnpk*^Tg-cre^, and *Hnrnpk*^Tg-cre^*/Tp53*^lox/WT^. (**B**) Representative images of HPG assay and fluorescence intensity values (dot plot, >1.000 cells of representative well; biological replicate analysis, *Hnrnpk*^Tg-cre^ vs. *Hnrnpk*^Tg-cre^*/Tp53*^lox/WT^, *P* = 0.0006). Scale bar: 100 μm. (**C**) Representative confocal microscopy images of NCL (left), FBL (right) HNRNPK, and DAPI staining. For dot plot analysis of *Tp53*^lox/WT^, *Hnrnpk*^Tg-cre^, and *Hnrnpk*^Tg-cre^*/Tp53*^lox/WT^, Hnrnpk expression was measured with Alexa Fluor 488 intensity (dot plot cell replicates, >1.000; biological replicate analysis: *P* = 0.0044); NCL relocalization was analyzed with nucleoplasm Alexa Fluor 647 intensity (dot plot cell replicates, >1.000; biological replicate analysis: *P* = 0.0018); and FBL reload was assessed by Alexa Fluor 647 spot total area (dot plot cell replicates, >1.000; biological replicate analysis: *P* = 0.0038). Scale bar: 10 μm. (**D**) Representative cell cycle FCM histogram plots in *Hnrnpk*^Tg-cre^*/Tp53*^lox/WT^ (*n* = 4) vs. *Hnrnpk*^Tg-cre^*/Tp53*^lox/WT^ (*n* = 3) MEFs and bar graph of the percentage of G_1_, S and G_2_/M % (*P* = 0.02) from FCM analysis. (**E**) Bright-field microscope images of SA-β-galactosidase staining in *Hnrnpk*^Tg-cre^ vs. *Hnrnpk*^Tg-cre^*/Tp53*^lox/WT^ MEFs and dot plot of cells positive for SA-β-galactosidase staining: *Hnrnpk*^Tg-cre^*/Tp53*^lox/WT^ (*n* = 8) vs. *Hnrnpk*^Tg-cre^ (*n* = 6) (*P* = 0.0007). All graphs are shown as the median or mean. All 2-group statistical analyses were 2-sided Student’s *t* test; for 3-group statistical analyses, 2-way ANOVA and Dunn’s multiple comparisons test were used: *P < 0.05; **P < 0.01; ***P < 0.001. All experiments comprised at least *n* = 3 biological replicates and/or *n* = 3 technical replicates.

To confirm the dependency of the nucleolar stress response in our model, we developed MEFs with HNRNPK overexpression and NCL knockdown (*Hnrnpk^Tg-CreERT2^/Ncl*^Kd^) (Supplemental Figure 3A) or with p53 haploinsufficiency (*Hnrnpk^Tg-cre^/Tp53*^lox/WT^) (Figure 4A). On the one hand, NCL haploinsufficiency reversed some of the nucleolar stress hallmarks (Supplemental Figure 3, B–D). On the other hand, p53 haploinsufficiency successfully rescued the protein synthesis rate (Figure 4B) and reverted the nucleolar stress hallmarks observed (Figure 4C), decreased cell cycle arrest (Figure 4D) and senescence (Figure 4E), and recovered the cell phenotype. These results confirmed the dependency of p53 on nucleolar driven by HNRNPK.

### HNRNPK-overexpressing HSCs are dysfunctional.

**Figure 5:**
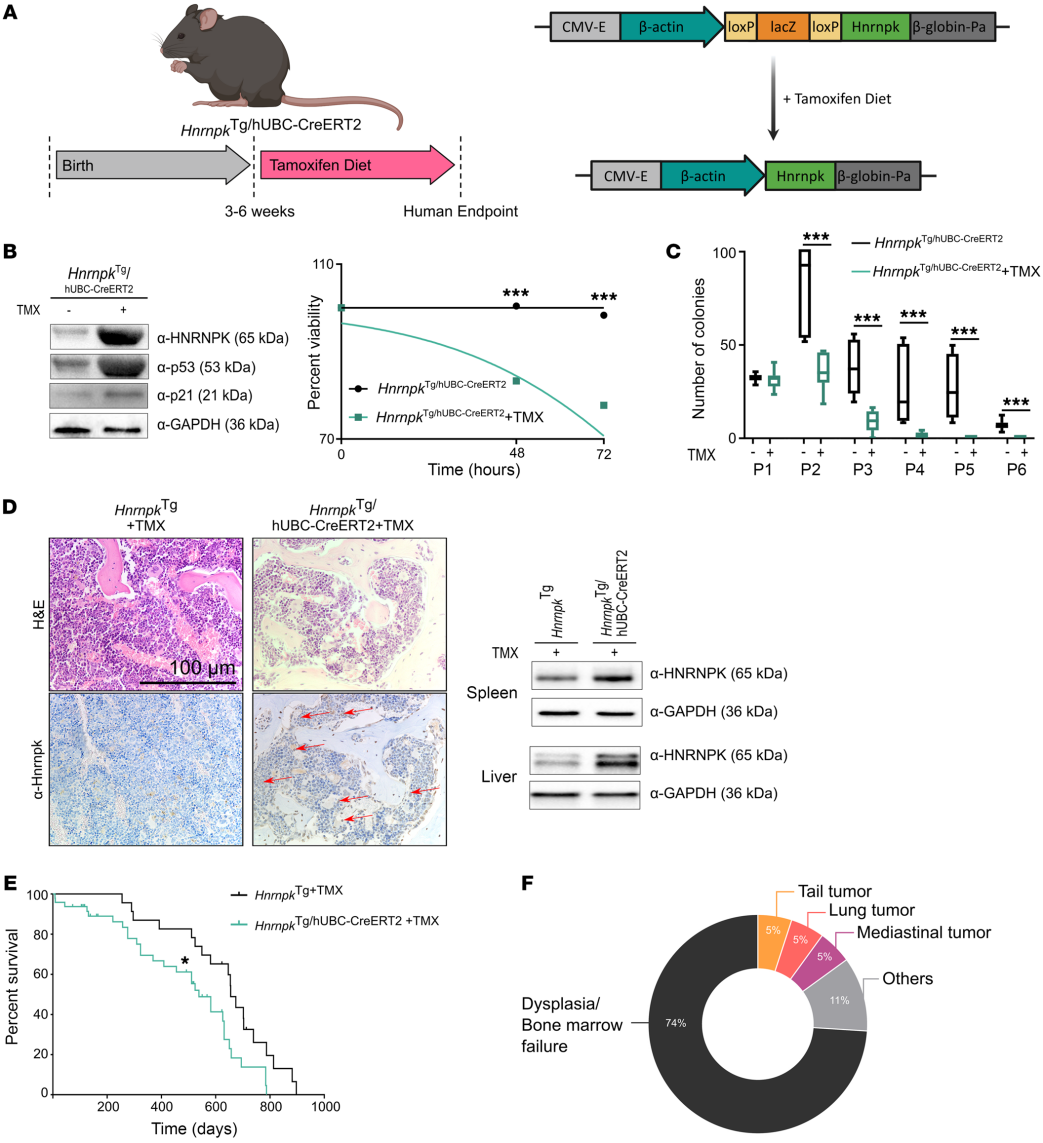
HSCs of the *Hnrnpk*^Tg/hUbc-CreERT2^ mouse model are dysfunctional. (**A**) Experimental design, model description, and flow chart for the *Hnrnpk*^Tg/hUbc-CreERT2^ mouse model. (**B**) Left: Western blot membrane of *Hnrnpk*^Tg/hUbc-CreERT2^ HSCs with and without (control) tamoxifen (TMX) showing HNRNPK overexpression and increases in p53 and p21. Right: Percentage viable *Hnrnpk*^Tg/hUbc-CreERT2^ HSCs with and without (control) TMX measured after 48 hours and 72 hours of short-term culture (*Hnrnpk*^Tg/hUbc-CreERT2^ = 7, control = 7, 48 hours, *P* = 0.0001; 72 hours, *P* = 0.0001). (**C**) Colony formation unit cultures from 6 passages of the replating assay of *Hnrnpk*^Tg/hUbc-CreERT2^ HSCs with (*n* = 8) and without (control, *n* = 8) TMX (P2, *P* = 0.0002; P3, *P* = 0.0002; P4, *P* = 0.0002; P5, *P* = 0.0002; P6, *P* = 0.0002). Arrows indicate positive cells. (**D**) Bone marrow HNRNPK IHC from TMX-induced *Hnrnpk*^Tg/hUbc-CreERT2^ mice and HNRNPK Western blot membrane of TMX-induced *Hnrnpk*^Tg/hUbc-CreERT2^ liver and spleen tissues. Scale bar: 100 μm. (**E**) Kaplan-Meier survival curve of *Hnrnpk*^Tg/hUbc-CreERT2^ (TMX) vs. *Hnrnpk*^Tg^ (TMX) mice (*Hnrnpk*^Tg/hUbc-CreERT2^ = 49, *Hnrnpk*^Tg^ = 22; *P* = 0.032; HR, 0.5305). (**F**) Pie chart with cause of death/phenotype developed for *Hnrnpk*^Tg/hUbc-CreERT2^ mice (*n* = 14). All graphs are shown as the mean ± SD. All images are representative of at least *n* = 3 mice and 4 random pathological areas. All experiments comprised at least *n* > 2 biological replicates and/or *n* > 3 technical replicates.

To study the impact of HNRNPK overexpression in an in vivo context, we developed *Hnrnpk*^Tg^ mice. To overcome potential developmental issues caused by HNRNPK (17), we used a 4-OHT–inducible hUBC^CreERT2^ model (Figure 5A).

We cultured HSCs from *Hnrnpk^Tg-hUBC-CreERT2^* total bone marrow and confirmed HNRNPK overexpression after induction, with a molecular pattern similar to that of *Hnrnpk*^SAM^ cells (Figure 5B). We phenotypically characterized these cells and observed a decrease in their viability (Figure 5B). Moreover, these HSCs had quicker exhaustion rate (Figure 5C and Supplemental Figure 4A) and a reduction in CD34^+^ cells in the culture (Supplemental Figure 4B), consistent with a prosenescent HSC profile. We confirmed that the activated *Hnrnpk^Tg-hUBC-CreERT2^* mouse model showed detectable HNRNPK overexpression of in hematopoietic tissues, as determined by IHC and/or Western blotting (Figure 5D).

### The HNRNPK-overexpressing mouse model shows bone marrow failure, lifespan reduction, and an aging phenotype partially rescued by p53 haploinsufficiency.

We observed a reduction in lifespan of our *Hnrnpk^Tg-hUBC-CreERT2^* mouse model (Figure 5E), which could be mainly attributed to dysplasia and/or bone marrow failure (Figure 5F), as well as different signs of aging: skeletal deformities and/or severe kyphosis, with white and/or bald hair patches (Supplemental Figure 4C and Table 1).

**Figure 6:**
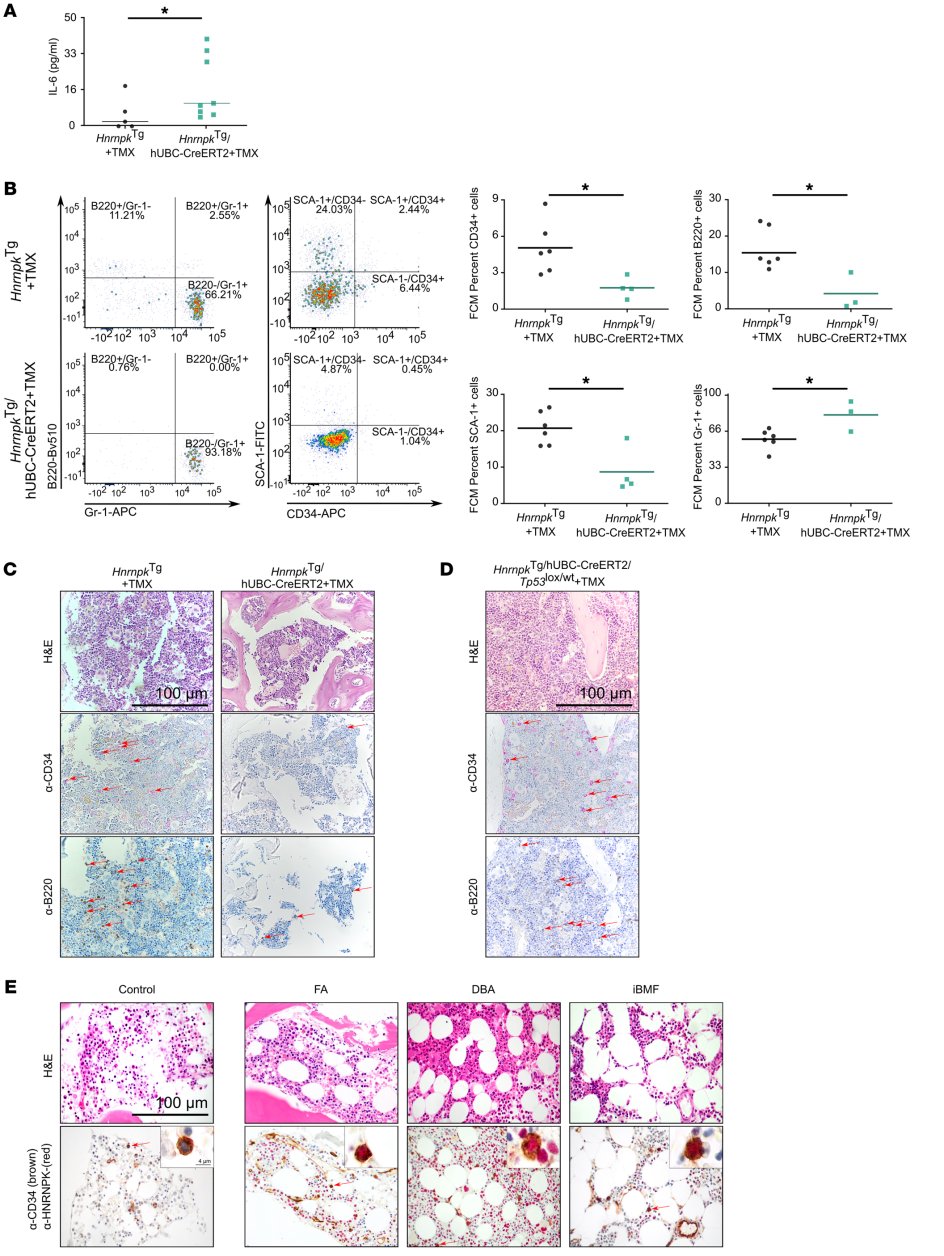
hnRNP K overexpression drives bone marrow failure in vivo. (**A**) In vivo senescence analysis by ELISA IL-6 dot plot concentration (TMX-induced and age-paired Hnrnpk^Tg/hUbc-CreERT2^ mice = 8 vs. Hnrnpk^Tg^ mice = 5; *P* = 0.020). (**B**) Representative sample FCM dot plot of CD34/SCA-1 (*Hnrnpk*^Tg/hUbc-CreERT2^ mice = 4 vs. *Hnrnpk*^Tg^ mice = 6; CD34^+^, *P* = 0.0190; SCA-1^+^, *P* = 0.0381) and Gr-1/B220 (*Hnrnpk*^Tg/hUbc-CreERT2^ = 3 vs. *Hnrnpk*^Tg^ = 6; Gr-1^+^, *P* = 0.0476; B220^+^, *P* = 0.0238) and box plot analysis of total bone marrow from TMX age-paired at 300 days. (**C**) HSC and lymphoid cell IHC analysis in bone marrow from TMX-induced *Hnrnpk*^Tg/hUbc-CreERT2^ mice, with H&E, CD34, and B220 staining. Scale bar: 100 μm. (**D**) HSC and lymphoid cell IHC analysis in bone marrow from TMX-induced *Hnrnpk*^Tg/hUbc-CreERT2^*/Tp53*^lox/WT^ mice. Red arrows indicate positive cells. Scale bar: 100 μm. (**E**) HNRNPK (brown) and CD34 (red) IHC analysis in bone marrow from clinical samples of individuals acting as controls (absence of bone marrow failure) and patients with Fanconi anemia (FA), Diamond-Blackfan anemia (DBA), and aplastic anemia (iBMF). Scale bar: 100 μm.

We also observed higher levels of the prosenescent cytokine IL-6 in peripheral blood serum, measured by ELISA, consistent with HSC exhaustion and aging phenotype (Figure 6A).

When analyzing the bone marrow by H&E, IHC, and flow cytometry (FCM) (gate strategy shown in Supplemental Figure 6D), we observed several abnormalities, including a reduction in lymphoid B220^+^ cells (Figure 6, B and C), a higher proportion of myeloid lineage Gr1^+^ (Figure 6B) and Mpo^+^ (Supplemental Figure 6E) cells, and impaired hematopoiesis with a reduction in CD34^+^ (Figure 6, B and C) and Sca1^+^ cells (Supplemental Figure 6E).

Finally, we confirmed the partial rescue of bone marrow failure and HSC exhaustion in vivo by p53 haploinsufficiency, observing a recovery of HSC CD34^+^ and lymphocyte B220^+^ cells (Figure 6D and Table 2) in these animals.

In summary, HNRNPK overexpression in vivo induces a ribosomopathy-like phenotype with bone marrow failure, caused by a prosenescent phenotype of HSCs via p53.

### Patients with ribosomopathies that cause bone marrow failure overexpress HNRNPK.

Finally, we aimed to analyze the expression of HNRNPK in different ribosomopathies leading to bone marrow failure.

HNRNPK protein levels were found to be elevated in the HSCs (CD34^+^) (21 of 27, 78%) from patients with Fanconi anemia (10 of 11, 91%); Diamond-Blackfan anemia (6 of 8, 75%), and aplastic anemia (5 of 8, 62%), with 50% HNRNPK^+^ cells and greater than grade 2 intensity when compared with the bone marrow samples from the control group (4 of 13, 30%). In this group, 70% of samples studied had low HNRNPK expression: less than 50% HNRNPK^+^ cells and/or lower than grade 1 intensity (Figure 6E, Table 3, and Supplemental Table 3). This result suggests that, even in cases in which HNRNPK is not a driver of bone marrow failure, patients present high HNRNPK levels, and this may contribute to disease progression in patients with ribosomopathies.

## Discussion

Here, we identify HNRNPK as a regulator of nucleolar-ribosome crosstalk, where upregulation of this RBP alters RiBi, leading to nucleolar abnormalities. These alterations cause an impairment in translation, inducing senescence and disrupting correct hematopoiesis, which causes and/or contributes to a ribosomopathy phenotype.

HNRNPK overexpression results in dysregulation of RiBi through NCL (26), thus inhibiting the expression and altering the synthesis and processing of ribosome components such as rRNAs (Figure 3, A–D). This ribosome imbalance correlates to an interruption of mRNA translation (27–29) (Figure 1, D and E). Additionally, HNRNPK upregulation alters the nucleolus composition and structure, leading to nucleolar stress (Figure 2A–C and Supplemental Figure 1C), with the consequent activation of p53, cellular arrest, and senescence (Figure 3, F–H). In mammals, the response to nucleolar stress is p53 dependent, in line with our observations of the nucleolar stress phenotype caused by HNRNPK overexpression, since p53 haploinsufficiency is able to rescue the phenotype in vitro (Figure 4, B and C).

Recently, evidence has correlated the occurrence of nucleolar stress with accelerated aging phenotypes and ribosomopathies in adult mammals (30). Similarly, in our in vivo model, HNRNPK overexpression had an effect on the lifespan, aging, and hematopoiesis of mice, showing a ribosomopathy-like phenotype. We have conclusive data indicating that HNRNPK-overexpressing cells show hallmarks of aging (31) (Figure 5, B and C). Indeed, one of the most frequent signs of aging is inefficient hematopoiesis, leading to bone marrow failure, similar to that in our mouse model (Figure 6, A–C). Our in vitro data strongly suggest that HNRNPK can reduce the mouse lifespan owing to nucleolar disruption and stress. It is known that the levels of FBL and NCL alter nucleolar structure and are correlated with prolonged or reduced metabolism and lifespan. As such, FBL haploinsufficiency leads to smaller nucleoli and is correlated with prolonged lifespan, while NCL haploinsufficiency leads to the opposite (4). However, during nucleolar stress, NCL was increased and delocalized, and FBL decreased, causing the halt of RiBi and function (Figure 1E, Figure 2B-C and Supplemental Figure 2E). This evidence proves that it is not the size of nucleoli or the metabolism and ribosome activity per se that correlate with lifespan but a proper functionality and balance between both the ribosome and nucleolar components.

Alteration of nucleolar and ribosomal components lead to p53 activation, which, in the context of HSCs, promotes arrest and senescence, initiating their exhaustion. This is evidenced by rescuing the molecular mechanism in vitro and the correct hematopoiesis by p53 in vivo.

Finally, the results from samples from patients with ribosomopathies confirm that HNRNPK and the consequent molecular cascade is a pathway commonly overexpressed in these syndromes, contributing to their pathophysiology.

We show a mechanism that triggers ribosomopathies: a gain of expression of HNRNPK. Its overexpression is enough to dysregulate RiBi and interrupt its activity, as well as nucleolar stress, altogether leading to an increase in p53, senescence, and bone marrow failure. This is in contrast with the currently accepted paradigm that it is the loss of ribosomal molecules that causes these syndromes. However, our data highlight that the subjacent cause of ribosomopathies is the unpropelled RiBi, regardless of being driven by a loss in a ribosomal component or, as in this case, by dysregulation of a master regulator. Although future work is needed to identify whether other RBPs can lead to a similar phenotype, we here identify HNRNPK as a driver of nucleolar stress, RiBi disruption, and ribosomopathy phenotype both in vitro and in vivo.

## Methods

### Sex as a biological variable.

Both female and male patients and mice were considered in this study, and sex was taken into account as a biological variable.

### Patients.

A total of 48 human patient samples were analyzed. We gathered 32 samples from patient diagnosed with bone marrow failure, including inherited bone marrow failure syndromes: 13 with Fanconi anemia (FA), 8 with Diamond-Blackfan anemia; 11 with aplastic anemia and 16 samples from age-paired individuals without bone marrow failure who acted as controls: 4 with solid tumors without bone marrow infiltration (3 with neuroblastoma, 1 with Ewing sarcoma), 1 with solid tumor with infiltration (neuroblastoma), 1 with infiltrated acute lymphoblastic leukemia, 1 with noninfiltrated acute lymphoblastic leukemia, 2 with infiltrated Burkitt lymphoma, 1 with infiltrated acute myeloid leukemia, and 6 with idiopathic thrombocytopenic purpura. Patients were diagnosed and samples collected at Hospital Infantil Universitario Niño Jesús. Samples were sent between March 2023 and January 2024 to Hospital Universitario 12 de Octubre for analysis. Formalin-fixed bone marrow biopsies were diagnostically evaluated by clinical pathologists in the Department of Hematopathology at Hospital Niño Jesus. IHC was performed at Hospital 12 de Octubre as previously described (3), using antibodies against hnRNP K (ab39975, Abcam, 1:7,000) with Bond Polymer Refine Red Detection (D59390, Leica Biosystems red staining) and CD34 (PA0212, Leica Biosystems, 1:100) with Polymer Refine Detection (DS9800, Leica Biosystems, brown staining). Two independent pathologists scored the HNRNPK expression as of a percentage of HNRNPK positivity in CD34^+^ cells, which was divided into 3 grades of HNRNPK intensity. Over 10 cells for each case were analyzed, and pathologists were blind to the clinical outcomes. Disagreements were resolved by joint review on a multihead microscope.

### HNRNPK-overexpressing animal model generation.

To generate a conditional HNRNPK-overexpressing model, we cloned full-length *Hnrnpk* as a BglII-XhoI fragment into the *pCALL2* vector. This construct contains a chicken β-actin promoter with an upstream cytomegalovirus enhancer (pCAGGS). This promoter is followed by a loxP-flanked LacZ/neoR fusion with 3 SV40 polyadenylation signals and the full-length *Hnrnpk* cDNA. We refer to the expression vector as *pCALL2-Hnrnpk*. The linearized vector was injected into blastocysts to generate chimeric mice by the Genetically Engineered Mouse Facility at the MD Anderson Cancer Center. Transgenic mice were mated with C57BL/6 mice to produce transgenic offspring. The *pCALL2-Hnrnpk* transgene was genotyped by real-time PCR on DNA extracted from tail clips.

*Hnrnpk^Tg^* mice were crossed with a mouse strain carrying ubiquitously expressed, tamoxifen-activated recombinase, hUBC-CreERT2, to generate *Hnrnpk^Tg^* and *Hnrnpk^Tg-hUBC-CreERT2^* mice. These mice were fed ad libitum with a 4-hydroxytamoxifen-containing diet (4-OHT, Teklad, 130856) until sacrifice, starting at 3–6 weeks of age.

The *Hnrnpk^Tg-hUBC-CreERT2^/Tp53^lox/WT^* model was generated by mating *Hnrnpk^Tg-hUBC-CreERT2^ mice* with *Tp53^lox/lox^* mice from the CNIO Animal facility.

### Mouse genotyping and validation of the Hnrnpk allele and p53_lox.

PCR-based strategies using primer sets that were external and internal to both the 5′ and 3′ arms were initially performed to confirm homologous recombination and germline transmission. The 5′ arm was verified by PCR amplification and visualization using external and internal primers. The *pCALL2*-*Hnrnpk* transgene genotyping was performed by real-time PCR allelic discrimination on genomic DNA at the CNIO Mouse Genome Editing Core Unit service with the following primers and hydrolysis probe: *Hnrnpk*, F_30F12 CCAGATACAGAACGCACAGT; *pCALL*, R_30F13 AAGGGGCTTCATGATGTCC; and *pCALL*, S_30F14 Fam-CTCGAGGTGGCTGCGATC-Zen-IBFQ. For *Tp53-*flox genotyping, we used p53Flox-F_1F10, GGAATACTTCAAGAGACGGAGA; p53Flox-R_1F11, AGCCAGGACTACACAGAGAA, and probes p53Flox-WT_1F13, Hex-CAAATTATGATTCGAACAGAATAAAGGATT-Zen-IBFQ, and p53Flox-lox_1F12, Fam-CTGCAGATAACTTCGTATAGCATACAT Zen-IBFQ.

### Pathological analysis and IHC.

Tissue samples were fixed in 10% neutral buffered formalin (4% PFA in solution), paraffin embedded and cut at 3 μm, mounted in TOMO slides, and dried overnight. For different staining methods, slides were deparaffinized in xylene and rehydrated through a series of graded ethanol and then water. Consecutive sections were stained with H&E, and several IHC reactions were performed in an automated immunostaining platform (Autostainer Link 48, Dako; Ventana Discovery XT, Roche).

Antigen retrieval was first performed with the appropriate pH buffer (low or high pH buffer, Dako; CC1m, Ventana, Roche), and endogenous peroxidase was blocked (3% peroxide hydrogen). Then, slides were incubated with the appropriate primary antibody as detailed in Supplemental Table 6.

After primary antibody incubation, slides were washed and incubated with the corresponding secondary antibodies conjugated with horseradish peroxidase.

Immunohistochemical reactions were developed using 3, 30-diaminobenzidine tetrahydrochloride (Chromo Map DAB, Ventana, Roche, #760-159), and nuclei were counterstained with Carazzi’s hematoxylin. Finally, the slides were dehydrated, cleared, and mounted with permanent mounting medium for microscopic evaluation.

Positive control sections known to be primary antibody positive were included for each staining run.

For human samples, we performed a sequential double staining with CD34 (Bond Polymer Refine Detection, Leica Biosystems, DS9800, brown staining) and HNRNPK (Bond Polymer Refine Red Detection, Leica Biosystems, D59390, red staining). Unmasking for both antibodies was at pH 6 for 20 minutes.

### IL-6 ELISA quantification.

Peripheral blood of euthanized mice was left to clot, and serum was frozen at –80°C until measurement. ELISA was used to determine levels of secreted IL-6 (Mouse IL-6 DuoSet ELISA Kit, R&D, DY406). Measurement and analysis of IL-6 levels were carried out according to the manufacturers’ instructions.

### MEF cultures.

MEFs from WT or *Hnrnpk*^Tg/Ella-Cre^ and *Hnrnpk*^Tg/UBC-CreERT2^ mice were obtained from 12.5-day-old embryos. Tissue was dissociated using mechanical and enzymatic digestion, with trypsin (Gibco, 25300096). The resulting cells were cultured in Dulbecco’s modified Eagle’s medium (Sigma-Aldrich, D5796) supplemented with 10% FBS (Sigma-Aldrich, F7524) and 5 μg/mL penicillin/streptomycin (P/S, Solmeglas, SOPENSRP), and cultured at 37°C and 5% CO_2_.

### Generation of HNRNPK-overexpressing MEFs (mouse derived and CRISPR/SAM edited).

MEFs derived from *Hnrnpk*^Tg/Ella-Cre^ constitutively overexpress HNRNPK. To achieve overexpression in *Hnrnpk*^Tg/UBC-CreERT2^ MEFs, treatment with 1 μM 4-OHT (Sigma-Aldrich, H7904) for 24 hours was needed. To generate HNRNPK-overexpressing cellular models, WT MEFs were transduced with lentiviral particles containing the plasmids lentiMPHv2 (Addgene, 89308) and lentiSAMv2 (Addgene, 75112) containing the sgRNAs to activate *Hnrnpk*. These guides were designed using the CRISPR-ERA design tool (http://crispr-era.stanford.edu/) and were cloned and inserted into lentiSAMv2 as previously described (32, 33). At 72 hours after transduction, cells were selected with 2 μg/mL blasticidin (AG Scientific, USA, B1247) and 400 μg/mL hygromycin (Roche, 10843555001) for 7 days. The surviving cells were referred to as MEFs-dCas9. The correct insertion of the sgRNA sequences was confirmed by Sanger sequencing (performed at the Genomics Unit at CNIO). The plasmid lentiSAMv2 with a nontargeting sgRNAs was used as a control. Lentiviral particle production was performed as previously described (34). Lentiviruses were used to infect 3 × 10^5^ cells cultured in medium supplemented with 8 μg/mL polybrene (Santa Cruz, sc-134220) and seeded in a 6-well plate. To mitigate possible biases due to off-target effects of the sgRNAs, *Hnrnpk*^SAM^ models were generated using two different sgRNAs per gene. The sequences of the sgRNAs were as follows: (a) sg*Hnrnpk*1: CACCGCGCTGCTCACGTGTGCCGGG; (b) sg*Hnrnpk2*: CACCGCCGAGGGAGTTTGGCGCGAT; and (c) sgNon-Targeting (sgNT): CACCGCCGAGGGAGTTTGGCGCGAT.

We used the guides independently, not in combination. The expression of *Hnrnpk* was quantified with qRT-PCR, and HNRNPK protein was measured by Western blot.

### HSC cultures.

A total of 1 × 10^6^ mononuclear bone marrow cells (as a source of CD34^+^ HSCs) were extracted from *Hnrnpk*^Tg/UBC-CreERT2^ mice. After extraction, they were cultured using StemSpan SFEM (STEMCELL Technologies, 09600) media supplemented with 10 μg/mL mTpo and 10 μg/mL mScf (PeproTech, 315-14 and 250-03). After 24 hours, fresh medium was added. All cells were maintained at 37°C and 5% CO_2_. After 72 hours of culture, cells were ready to use in viability assays. Briefly, 1 × 10^5^ cells/mL were seeded with and without 10 μg/mL 4-OHT, and after 48–72 hours, WST-1 assay was used according to the manufacturer’s protocol (Sigma-Aldrich, 11644807001).

### Colony formation replating assays.

A total of 4 × 10^5^ bone marrow cells from *Hnrnpk*^Tg/UBC-CreERT2^ mice were seeded in MethoCult methylcellulose medium supplemented with cytokines (STEMCELL Technologies, GF M3434) with and without 10 μg/mL 4-OHT. Fourteen days after seeding, viable cells were counted using trypan blue, and 4 × 10^5^ cells were reseeded in duplicate cultures. This process was repeated on a 14-day cycle for 6 cycles.

### RNA-Seq analysis of HNRNPK-overexpressing MEFs.

Total RNA samples from empty vector and *Hnrnpk*^SAM^ MEFs were converted into sequencing libraries with the NEBNext Ultra II Directional RNA Library Prep Kit for Illumina (NEB, E7760), as recommended by the manufacturer. Briefly, the polyA^+^ fraction was purified and randomly fragmented, converted to double-stranded cDNA, and processed through subsequent enzymatic treatments of end-repair, dA-tailing, and ligation to adapters. The adapter-ligated library was completed by PCR with Illumina PE primers. The resulting purified cDNA libraries were applied to an Illumina flow cell for cluster generation and sequenced on a NextSeq500 (Illumina) instrument in a paired-end 43+43 base format by following the manufacturer’s protocols. The resulting reads were analyzed with the Nextpresso pipeline as follows: (a) Sequencing quality was checked with FastQC v0.11.0 (https://www.bioinformatics.babraham.ac.uk/projects/fastqc/) (35). (b) Reads were aligned to the mouse genome (GRCm39) with TopHat2 (36) using Bowtie2 (37) and SAMtools (38), allowing 3 mismatches and 20 multihits. (c) The Gencode vM29 gene annotation for GRCm39 was used. Read counts were obtained with HTSeq (39). (d) Differential expression and normalization were performed with DESeq2 (40), keeping only those genes for which the normalized count value was higher than 10 in at least 30% of the samples. (e) Finally, those genes that had an adjusted *P* value below 0.05 FDRs were selected. GSEAPre-ranked was used to perform gene set enrichment analysis for the selected gene signatures on a pre-ranked gene list, setting 1000 gene set permutations (21). Only those gene sets with significant enrichment levels (FDR *q* < 0.05) were considered. The data discussed in this publication have been deposited in NCBI’s Gene Expression Omnibus (GEO) (41) and are accessible through GEO Series accession GSE242038 (https://www.ncbi.nlm.nih.gov/geo/query/acc.cgi?acc=GSE242038).

### TMTpro analysis of HNRNPK-overexpressing MEFs.

Cells were lysed in 4% SDS in 50 mM TEAB at 95°C for 10 minutes. Protein concentration was determined using the BCA Protein Assay (Thermo Scientific, 23225) following manufacturer’s instructions. Proteins were reduced [15 mM tris(2-carboxyethyl)phosphine] and alkylated (30 mM 2-chloroacetamide) for 30 minutes in the dark at room temperature. Samples were digested in an automated Apex King Fisher instrument (Thermo Scientific). The resulting peptides were speed-vac dried and resuspended in 200 mM HEPES, pH 8.5. Subsequent peptides were labeled using TMT reagent 18-plex (Waters) following the manufacturer’s instructions. Samples were pooled equally based on total peptide amount, which was determined by comparing overall signal intensities on a regular LC-MS/MS run. The final mixture was finally desalted using a Sep-Pak C18 cartridge (Waters).

For total proteome analysis, labeled peptides were fractionated by means of high pH reversed-phase chromatography using an Ultimate 3000 HPLC system (Waters) equipped with a sample collector. A total of 45 fractions were collected at minute 15 of analysis; each fraction was recorded for 60 seconds and concatenated into 34 fractions.

LC-MS/MS analysis was performed by coupling an Ultimate 3000 RSLCnano System (Dionex) to an Orbitrap Exploris 480 mass spectrometer (Thermo Scientific). Peptides were loaded into a trap column (Acclaim PepMap 100, 100 μm × 2cm, Thermo Scientific, 164946) for 3 minutes at a flow rate of 10 μL/min in 0.1% FA. Then, peptides were transferred to an analytical column (PepMap RSLC C18, 2 μm, 75 μm × 50 cm, Thermo Scientific, 164946) and separated using a 60 minutes effective linear gradient (buffer A, 0.1% FA; buffer B, 100% ACN, 0.1% FA) at a flow rate of 250 nL/min. The peptides were electrosprayed (1.5 kV) into the mass spectrometer through a heated capillary at 300°C and a funnel RF level of 40%. Peptides were isolated using a 0.7 Th window and fragmented using higher-energy collisional dissociation at 36% normalized collision energy. The ion target values were 3 × 10^6^ for MS (25 ms maximum injection time) and 1 × 10^5^ for MS/MS (86 ms maximum injection time). Raw files were processed with MaxQuant (v 2.1.4.0) using the standard settings against a UniProt Reference mouse proteome (UniProtKB UP000000589, 21990 sequences downloaded on July 2019; https://www.uniprot.org/proteomes/UP000000589) supplemented with contaminants. The minimal peptide length was set to 7 amino acids, and a maximum of 2 tryptic missed-cleavages were allowed. The results were filtered at 0.01 FDR (peptide and protein level). The “proteinGroup.txt” file was loaded in the Prostar package (v1.22.3) (42) for further statistical analysis. Reporter intensities were log_2_ transformed and normalized using the Loess function from the Prostar package. Only proteins with a log_2_ ratio <–0.3 or >0.3 and *P* value <0.05 (FDR <5%) (Limma) were defined as regulated. The output was uploaded in Perseus (v1.6.7.0) for further analysis. GSEAPre-ranked analysis (v4.3.2) was performed on GO Terms.

The mass spectrometry proteomics data have been deposited to the ProteomeXchange Consortium via the PRIDE partner repository with the dataset identifier PXD046699.

### Immunofluorescence.

Cells were fixed with 4% PFA/PBS for 10 minutes at room temperature, permeabilized, and blocked with blocking buffer (0.5% BSA, 0.1% Triton X-100 in 1X PBS) overnight at 4°C. Then, samples were incubated overnight at 4°C with the appropriate concentration of primary antibodies diluted in blocking buffer. After washing with PBS, the corresponding secondary antibodies and 0.5 μg/mL DAPI (Invitrogen, D1306) were added overnight at 4°C in blocking buffer.

Images were analyzed within each cell. The total area of the nucleolus and number of dots were calculated by NCL measurement. The nucleolar stress signal was analyzed by the increase in NCL mean intensity in the nucleus of cells using DAPI as a counterstain. In these experiments, at least 20 pictures per well were taken using a ×20 water objective. Image analysis was performed using a homemade pipeline identifying the different organelles according to the intensity of the signal in the nucleus. Images for analysis were taken using two High Content Screening (HCS) systems, HCS Opera LX and HCS Opera Phenix Plus, and analyzed with their specific software: Acapella 2.6 (Perkin Elmer) and/or Harmony 5.1 (Perkin Elmer). Representative images were taken using a ×63 water objective using the HCS Opera Phenix Plus.

### Nucleolar stress assay and analysis.

MEFs (9,000 cells/well) were plated onto 96-well plates (Perkin Elmer, 6055300). The following day, the cells were either preexposed or not to 5 nM actinomycin D (Merck, A141050-76-0) for 4 hours. Then, the cells were fixed, blocked, and stained according to the previously described immunofluorescence protocol. Images were acquired as previously described (see *Immunoflorescence*).

Similarly, HSCs (40,000 cells/well) were plated onto 96-well plates (Perkin Elmer, 6055300) previously treated with poly-L-lysine solution (Merck, P8920) overnight at 4ºC. Once plated, cells were fixed, blocked, and stained according to the previously described immunofluorescence protocol. Likewise, images were acquired as previously described (see *Immunoflorescence*).

### Global transcription analysis by EU.

To quantify global RNA synthesis in vitro, we used the Click-iT RNA Alexa Fluor 488 kit (Thermo Fisher Scientific, C10329). MEFs (9,000 cells per well) were plated onto 96-well plates (Perkin Elmer, 6055300). The following day, cells were pulsed with EU for 60 minutes and then fixed with 4% PFA/PBS for 10 minutes at room temperature. Then, cells were permeabilized and blocked with blocking buffer (0.5% BSA, 0.1% Triton X-100 in 1X PBS) overnight at 4ºC. The detection was performed through the Click-iT chemistry reaction for 2 hours at room temperature, following manufacturer’s instructions. Finally, nuclei were counterstained with DAPI.

*Hnrnpk*^SAM^ MEFs were pulsed with EU as described above, while *Hnrnpk*^Tg-hUBC-CreERT2^ MEFs were pulsed with EU after different 1,000 nM 4-OHT induction times (1, 4, and 24 hours).

Immunofluorescence was detected and quantified using 2 HCS systems, HCS Opera LX and HCS Opera Phenix Plus and analyzed with their specific software, Acapella 2.6 (Perkin Elmer) and/or Harmony 5.1 (Perkin Elmer).

### Global translation analysis by HPG.

To detect and quantify newly synthesized proteins or changes in protein expression in vitro, we used the Click-iT L-homopropargilglicine assay Alexa Fluor 488 Protein Synthesis Assay Kit (Thermo Fisher Scientific, C10428). MEFs (9,000 cells per well) were seeded onto 96-well plates (Perkin Elmer, 6055300), and the following day they were pulsed with HPG for 90 minutes at 37ºC and 5% CO_2_, following fixation with 4% PFA/PBS for 10 minutes at room temperature. Then, cells were permeabilized and blocked with blocking buffer (0.5% BSA, 0.1% Triton X-100 in 1X PBS) overnight at 4ºC. After that, the detection was performed through Click-iT chemistry reaction over 90 minutes at room temperature, following the manufacturer’s instructions. Finally, nuclei were counterstained with DAPI. The same was done with HSCs, plating 55.000 cells/well, but in poly-L-lysine–treated p96 plaques as previously described (see *Immunoflorescence*).

Immunofluorescence was detected and quantified using 2 HCS systems, HCS Opera LX and HCS Opera Phenix Plus, and analyzed with their specific software, Acapella 2.6 (Perkin Elmer) and/or Harmony 5.1 (Perkin Elmer).

### Polysome profile assay.

Polysomal profiling analysis was performed by IMMAGINA BIOTECHNOLOGY. Briefly, cytoplasmic lysates were prepared by resuspending MEFs from 150 cm^2^ 70% confluent plates in 200 μL IMMAGINA lysis buffer (IMMAGINA BIOTECHNOLOGY, RL001-1). Cell suspension was passed through a 26-gauge needle 10 times, and cell debris and nuclei were pelleted by centrifugation at 20,000*g* for 5 minutes. Cleared supernatants were loaded on a linear 15%–50% sucrose gradient and ultracentrifuged in a SW41Ti rotor (Beckman) for 1.5 hours at 180,000*g* at 4°C in a Beckman Optima LE-80K ultracentrifuge. After ultracentrifugation, gradients were fractionated in 1 mL volume fractions with continuous monitoring absorbance at 254 nm using an ISCO UA-6 UV detector.

### Transmission electronic microscopy.

For Transmission electronic microscopy analysis, control (empty vector) and HNRNPK-overexpressing MEFs were fixed with 3% glutaraldehyde in 0.12 M phosphate buffer (pH 7.2) for 1 hour at room temperature. Cells were then scraped and centrifuged in a microfuge at 9,500*g* at room temperature for 10 minutes to obtain a pellet. After washing with 0.12 M phosphate buffer, pellets were post fixed in 2% osmium tetroxide at room temperature for 1 hour. Cell samples were then dehydrated in an acetone series and embedded in araldite resin (Durcupan ACM, Sigma-Aldrich). Ultrathin sections (50–60 nm) were obtained with an UltraCut UC7 ultramicrotome (Leica Microsystems), collected on Formvar-coated copper grids, and contrast stained with uranyl acetate and lead citrate. MEFs were examined with a JEM 1011 (JEOL) electron microscope operating at 80 kV. Micrographs were taken with a camera (Orius 1200A; Gatan) using Gatan microscopy suite (Gatan). Electron micrographs were processed using Adobe Photoshop (24.0.0).

### Flow cytometry analysis.

For cell cycle analysis, samples from MEF cultures were collected when 70% confluent and fixed with a glacial 70% ethanol solution overnight, followed by staining with 40 ng/mL DAPI overnight and incubation at 4°C. Then, the sample was passed through a 40 μm cell strainer to ensure single-cell suspension and analyzed using a FACS Canto II cytometer (BD Biosciences), which recorded 20,000 events per sample.

For immunophenotyping experiments, we analyzed the expression of LSK (c-KIT^+^/SCA1^+^), LT-HSC (CD34^–^/SCA-1^+^), ST-HSC (CD34^+^/SCA-1^+^), and myeloid-derived suppressor cells (MDSCs) (Gr-1^+^, CD11b^+^) and lymphoid (B220^+^), erythroid (Ter-119^+^), and megakaryocytic (CD41^+^) compartments using stem (HSC) and nonstem hematological cell panels. Samples were analyzed using an LSR Fortessa cytometer (BD Biosciences), which recorded more than 50,000 total events per sample. All data were analyzed with FCS Express 7 software.

### Senescence assays.

MEFs were plated onto 6-well plates until they reached 70%–80% confluence. Senescence-associated (SA) β-galactosidase activity was determined using the Senescence SA-β-Galactosidase Staining Kit (Cell Signaling, 9860) according to the manufacturer’s instructions. As senescent molecular markers such as p21 and p16 are expressed at low levels under steady-state conditions, we irradiated MEFs (*Hnrnpk*^SAM^) with 5 Gy and determined the levels of p21 and p16 protein with and without IR by Western blotting.

### Western blot.

Tissues and cells were homogenized in RIPA lysis buffer (Millipore, 20-188) containing protease (Complete Mini, Roche, 11836153001) and phosphatase inhibitors (PhosSTOP Roche, 4906837001). Soluble proteins were boiled in Laemmli buffer (Bio-Rad, 1610747) with β-mercaptoethanol, resolved on a 4%–20% gradient SDS-PAGE gel, and transferred to a PVDF membrane. Membranes were blocked in 5% nonfat milk for 1 hour at room temperature and then incubated with primary antibodies overnight at 4°C. After primary antibody incubation, membranes were washed in T-TBS and incubated with the corresponding HRP-conjugated secondary antibody to be detected by the enhanced chemiluminescence SuperSignal West Femto Maximum Sensitivity Substrate (Thermo Scientific, 34095). β-ACTIN and/or GAPDH were used as cellular loading controls.

### Northern blot.

Total RNA was isolated from murine cell lines using the Quick RNA Miniprep Kit (Zymo Research) according to the manufacturer’s protocols. A total of 4 μg RNA per lane was resolved on a 1% formaldehyde denaturing agarose gel. Northern blot analysis was conducted as previously described (43), and quantification according to the Rapid Analysis of Multiple Precursors method (44) is included in the Supporting Data Values file. The following murine biotin-labeled probes were used: ITS1-132, 5′-Biotin-TTCTCTCACCTCACTCCAGACACCTCGCTCCACA-3′ (45) or ITS2-2, 5′-Biotin-ACTGGTGAGGCAGCGGTCCGGGAGGCGCCGACG-3′ (46).

### qRT-PCR.

Total RNA from frozen tissue and cell cultures was extracted using the RNeasy kit (QIAGEN, 74134) following the manufacturer’s instructions. cDNA synthesis was performed using the iSCRIPT cDNA synthesis kit (Bio-Rad, 1708891) according to the manufacturer’s protocols. Quantitative real-time PCR was performed with QuantStudio 6 Flex (Applied Biosystems, Life Technologies) using SYBR qPCR master mix (Promega, 4367659).

*Gapdh* and/or *β-actin* served as housekeeping controls. Changes in expression were compared using the Pfaffl method (47) by comparing expression changes between target genes and the housekeeping controls.

### HNRNPK IP (IP and RNA Immunoprecipitation).

HNRNPK and associated proteins and RNAs were coimmunoprecipitated from *Hnrnpk*^SAM^ and empty vector MEFs using anti-HNRNPK (D6) antibody (Santa Cruz, sc-28380) coupled to Dynabeads M-270 Epoxy (Invitrogen, 14301). Coupling was performed following the manufacturer’s instructions, at a ratio of 7 μg primary antibody per 1 mg beads.

Cells were cryolized in liquid nitrogen, and samples were then resuspended in IP buffer. Samples were incubated with magnetic beads and isolated using a DynaMag-2 magnet (Invitrogen, 12321D).

### Statistics.

Statistical analyses comparing 2 groups were performed using unpaired 2-tailed *t* tests or Mann-Whitney tests (normal or nonnormal distribution, *P* < 0.05 in the normality KS test) for analysis between 2 groups. For dichotomous discrete variables, χ^2^ tests were performed (F-Fisher tests), and *P* < 0.05 were considered statistically significant. For multiple group analysis, a 2-way ANOVA Šidák’s and Dunn’s multiple comparisons tests were performed. To test differences in survival curves, the Kaplan-Meier test was used. Differences between survival distributions were analyzed using the log-rank test. Hazard ratios and confidence intervals were obtained by Mantel-Haenszel analysis. Statistical computations were performed using GraphPad Prism 7.0.

### Study approval.

The use of human samples was approved by the Comité Ético de Investigación Clínica of the Instituto de Investigación Biomédica of the Hospital 12 de Octubre, and all patients and donors provided written informed consent in accordance with the Declaration of Helsinki. The generation of the mouse model was conducted in the MD Anderson Genetically Engineered Mouse Facility with approval from their Institutional Animal Care and Use Committee under protocol 0000787-RN01. All mice were maintained at CNIO under specific pathogen–free conditions in accordance with the recommendations of the Federation of European Laboratory Animal Science Associations. All animal experiments were approved by the CNIO Ethical Committee (CEIyBA) under protocol PROEX158/18.

### Data availability.

The data discussed in this publication have been deposited in different repositories. The mass spectrometry proteomics data have been deposited to the ProteomeXchange Consortium via the PRIDE partner repository with the dataset identifier PXD046699 (https://www.ebi.ac.uk/pride/archive/projects/PXD046699). The RNA-Seq data have been deposited in NCBI’s Gene Expression Omnibus (41) and are accessible through GEO Series accession GSE242038 (https://www.ncbi.nlm.nih.gov/geo/query/acc.cgi?acc=GSE242038). We provide supporting data values associated with the manuscript and supplement material, including values for all data points shown in graphs and values behind any reported means, in the Supporting Data Values file.

## Author contributions

MG conceived and planned the study with input from SMP, PM, XZ, MJLA, and JML. PAG, MVE, MANA, AOS, AOR, and MAM designed and performed the culture, cellular, and molecular biology experiments as well as in vivo experiments. MIN designed and performed FCM experiments. MHS and RTR designed and performed the CRISPR-modified cellular models. AR, OB, and SMP performed Northern blotting. PAG, with help from DM, MP, GM, JG, OS, and VL, designed and performed the confocal microscopy experiments. ML designed and performed electron microscopy experiments. OD and OGC designed and performed the RNA-Seq experiments. EC and PJDA designed and performed the IHC mice experiments. JZ, JS, DA, RMGM, and ER obtained patient samples and clinical information and performed the IHC experiments. PXE and MI designed and performed TMTpro experiments. SRP and RTR designed and performed karyotyping experiments. MG wrote the manuscript with the contributions from MVE and PAG. MG supervised the study. All authors approved the final manuscript.

## Supplementary Material

Supplemental data

Supplemental tables 1-5

Supporting data values

## Figures and Tables

**Table 3:**
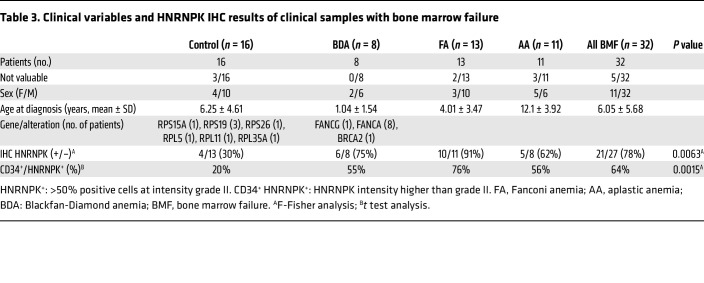
Clinical variables and HNRNPK IHC results of clinical samples with bone marrow failure

**Table 1:**
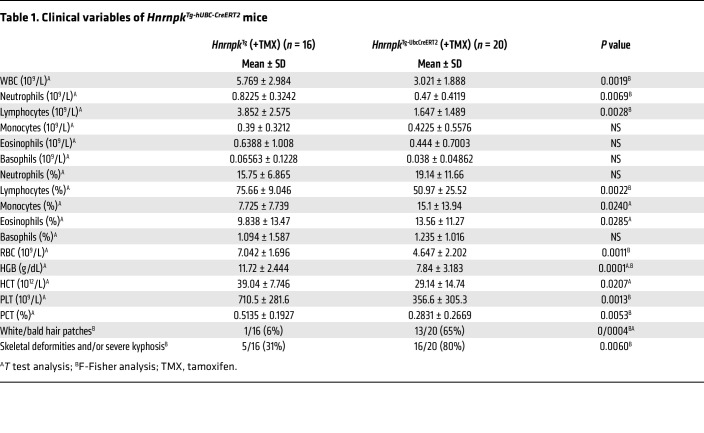
Clinical variables of *Hnrnpk^Tg-hUBC-CreERT2^* mice

**Table 2:**
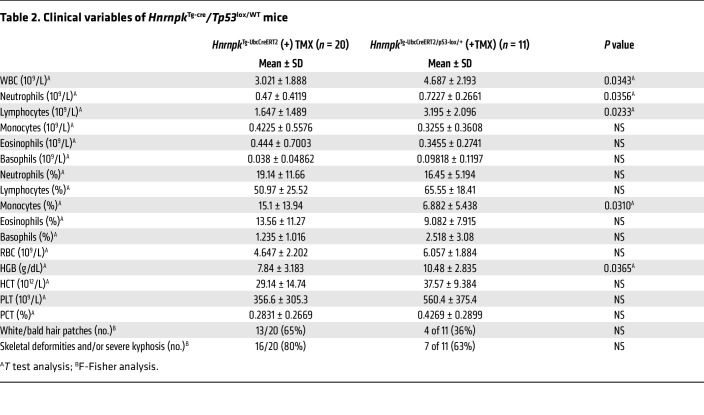
Clinical variables of *Hnrnpk*^Tg-cre^*/Tp53*^lox/WT^ mice

## Version 1 05/08/2025

In-Press Preview

## Version 2 06/16/2025

Electronic publication
