# Supplementary material for: The tumor suppressor HNRNPK induces p53-dependent nucleolar stress to drive ribosomopathies
Source: J Clin Invest. 2025 May 8;135(12):e183697. doi: 10.1172/JCI183697 (PMC12165811; doi:10.1172/JCI183697)
Supplement: Supplemental data [file jci-135-183697-s237.pdf]

Supplementary Materials for

The tumor suppressor HNRNPK induces p53-dependent nucleolar stress, driving ribosomopathies

Pedro Aguilar-Garrido *et al.*

Corresponding author: Miguel Gallardo Delgado, miguelgallardodelgado@gmail.com

The PDF includes:

Figs. S1 to S6

Key Resources Table

Supplementary Materials

Supplementary Figures and Tables

Aguilar-Garrido et al. Supplementary Material. Figure 1

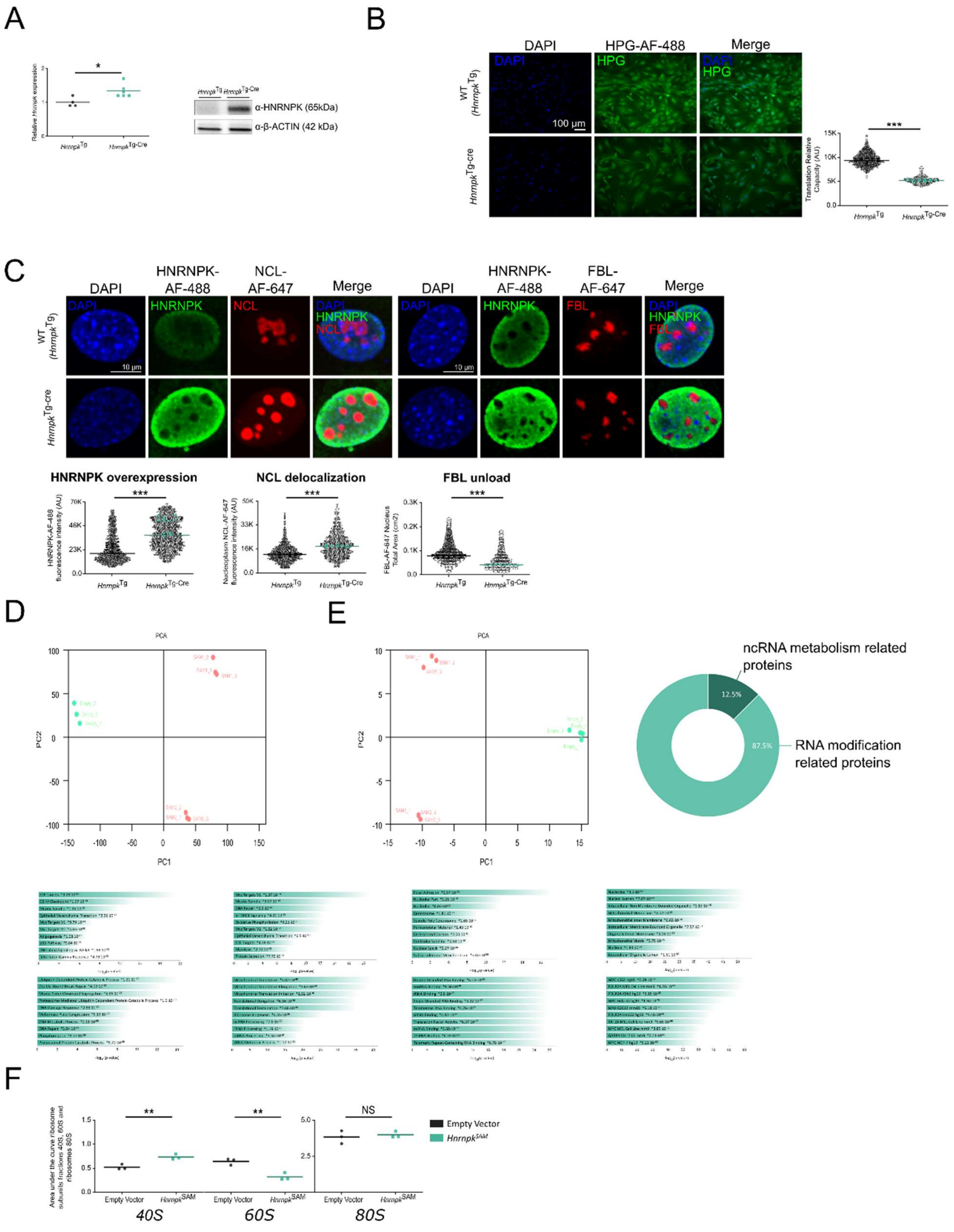

Fig. S1.

(A) Left: *Hnrnpk* qRT–PCR box-plot in *Hnrnpk*$^{Tg\text{-}cre}$ (p=0.022). Right: HNRNPK western blot membrane. (B) Representative images of HPG assay confocal microscopy in *Hnrnpk*$^{Tg\text{-}cre}$ vs WT. Right: Fluorescence intensity values from the HPG assay (dot-plot, >1.000 cells of representative well; p=0.0001, biological replicate analysis: p=0.0001). (C) Left: Representative confocal microscopy images with NCL, HNRNPK and DAPI. Right: Representative confocal microscopy images with FBL, HNRNPK and DAPI. Bottom: Representative confocal microscopy images of FBL, NCL and DAPI staining. (DAPI, nucleus, dot-plot cell replicates >1.000; biological replicates analysis: p=0.0001); HNRNPK expression: Alexa Fluor 488 intensity (HNRNPK, dot-plot cell replicates >1.000; biological replicates analysis: p=0.0001);; NCL nucleoplasm expression: Alexa Fluor 647 intensity (NCL delocalization, dot-plot cell replicates >1.000; biological replicates analysis: p=0.0001);; and FBL unload: Alexa Fluor 647 spot total area (FBL, dot-plot cell replicates >1.000: p=0.0001; biological replicates analysis: p=0.0001). (D) Representative Figs with the analysis of RNA-seq technique. Top: Principal component analysis of empty vector and *Hnrnpk*^{SAM} MEFs. Bottom. Enrichr analysis from top to bottom: Enrichr MsigDB hallmark 2020; GO Biological Process 2022; GO cellular components 2017b; GO Molecular Function 2017b. (E) Representative Figs with the analysis of TMTpro technique. Left: Principal component analysis of empty vector and *Hnrnpk*^{SAM} MEFs. Right: Pie-chart showing the percent distribution according to the function of downexpressed proteins in *Hnrnpk*^{SAM} MEFs. Bottom: Enrichr analysis from top to bottom: Enrichr MsigDB hallmark 2020; GO Biological Process 2021; GO cellular components 2021; Encode TF Chip-Seq 2015. (F) Ribosome subunits data dot-plot from the polysome assay (40S p=0.008; 60S p=0.005). Graphs are shown as the mean.

Aguilar-Garrido et al. Supplementary Material. Figure 2

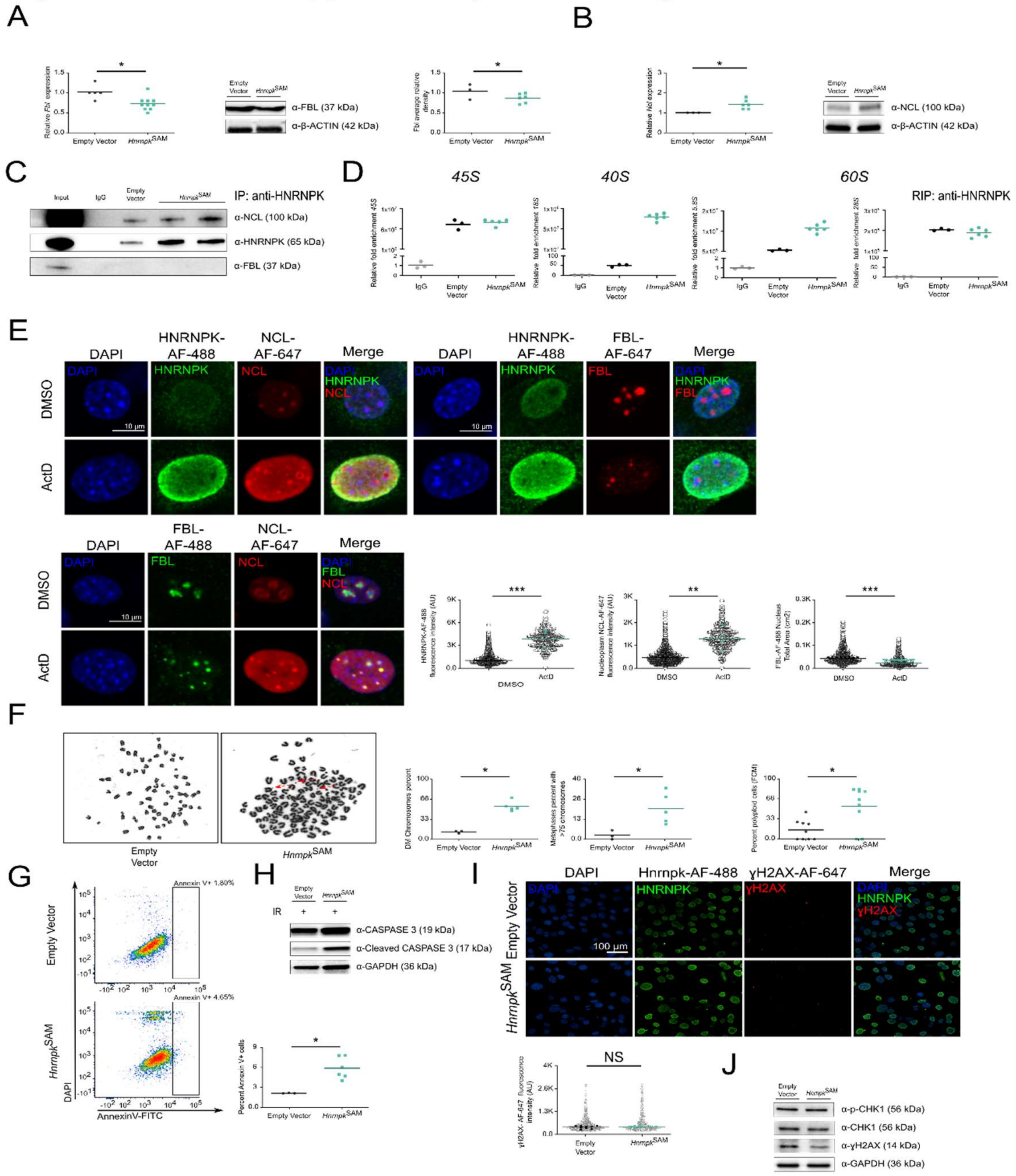

Fig. S2.

(A) Left: qRT–PCR results dot-plot showing *Fbl* expression (p=0.0117). Middle: FBL western blot membrane; Right: FBL western blot densitometry quantification dot-plot. (B) Left: *Hnrnpk*^{SAM} (n≥6) vs empty vector (n≥3) qRT–PCR results dot-plot showing *Ncl* expression (p=0.0119). Right: NCL western blots membrane (C) HNRNPK protein immunoprecipitation (IP) blot NCL and FBL immunedetection. (D) HNRNPK RNA

immunoprecipitation (RIP) analysis of rRNAs 45S, 28S, 18S and 5.8S. (E) Top Left: Representative confocal microscopy images of NCL, HNRNPK and DAPI staining in wild-type MEFs treated with ActD (5 nM). Top right: NCL, FBL and DAPI staining. Bottom: dot-plot analysis of Alexa Fluor 488 intensity (HNRNPK), Alexa Fluor 647 intensity (NCL) and Alexa Fluor 488 (FBL). Nucleoplasm Alexa Fluor 647 intensity (NCL delocalization) in MEFs treated with ActD (5 nM) (HNRNPK: dot-plot cell replicates >1.000; biological replicate analysis: p=0.0381;; NCL delocalization: dot-plot cell replicates >1.000; biological replicate analysis: 0.0095; FBL: dot-plot cell replicates >1.000: p=0.0001; biological replicate analysis: p=0.0001). Scale bar: 25μm. (F) Left: Representative microscope images of karyotype analysis. Middle: *Hnrnpk*^{SAM} (n=5) vs empty vector (n=3) MEF dot-plot quantification of metaphase percent with double minute (DM) (p=0.0357) and more than 75 chromosomes (p=0.0357). Right: Dot-plot with polyploidy (p=0.0151) from FCM analysis. (G) Left: Representative sample FCM DAPI/Annexin V-FITC staining dot plot analysis of *Hnrnpk*^{SAM} (n=6) vs empty vector (n=3) MEFs. Right: Dot-plot analysis of the Annexin V FCM assay (p=0.0238). (H) Western blot membrane of irradiated *Hnrnpk*^{SAM} showing an increase in the apoptosis marker cleaved caspase 3. (I) Confocal microscopy images of ɣH2AX, HNRNPK and DAPI staining in HNRNPK-overexpressing MEFs. Dot-plot analysis of representative well samples of: ɣH2AX Alexa Fluor 647 intensity (ɣH2AX, dot-plot cell replicates >1.000: p=NS; biological replicate analysis: p=NS). Scale bar: 100 μm. (J) p-CHK1, CHK1 and ɣH2AX western blots membrane. All graphs are shown as the mean (A, B, E & F) or median (D & H). Statistical analysis consisted of two-sided Student's t-test. All experiments comprised at least 2 biological replicates and/or 2 technical replicates.

Aguilar-Garrido et al. Supplementary Material. Figure 3

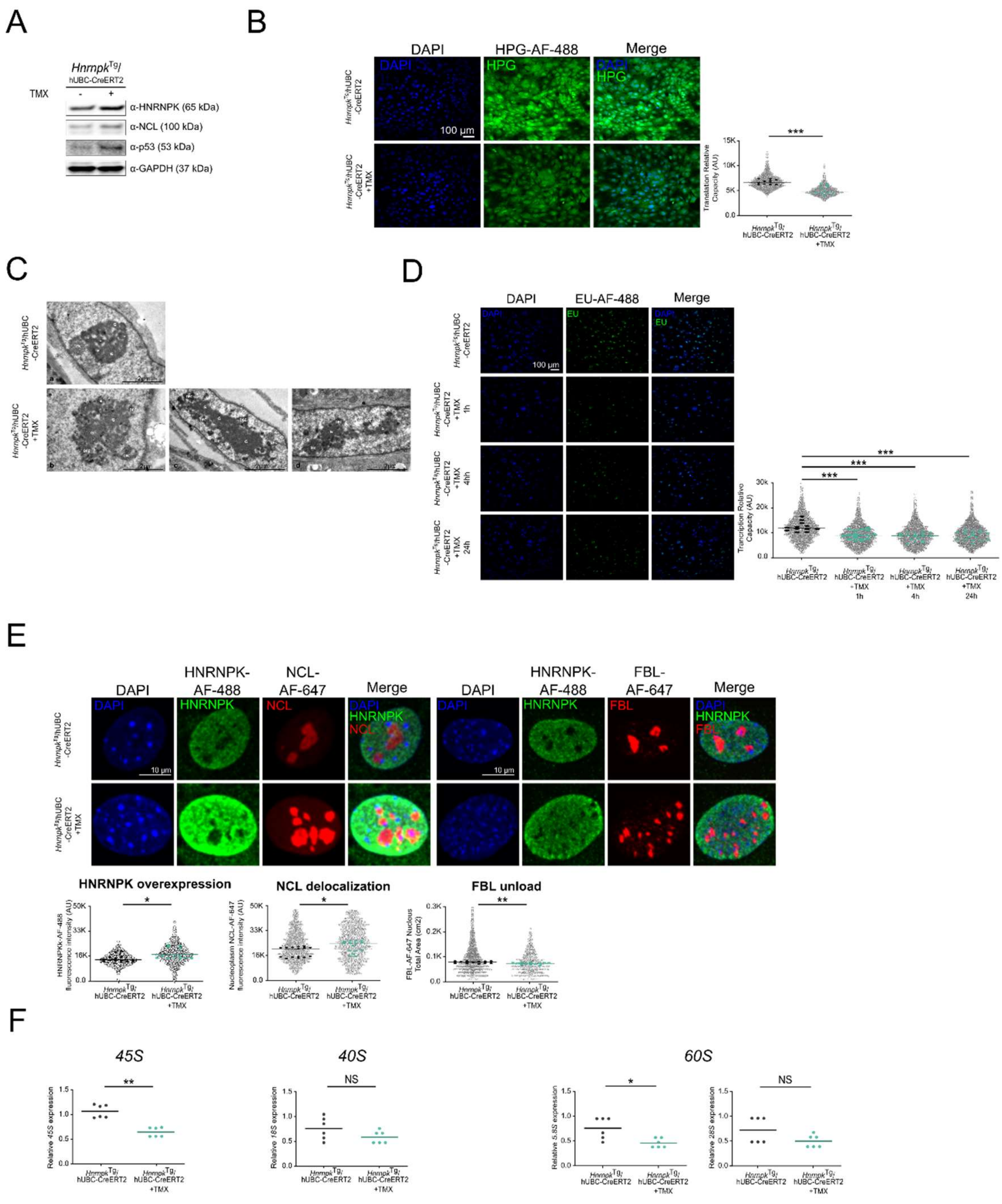

Fig. S3.

(A) Western blot membrane showing HNRNPK, p53 and NCL in *Hnrnpk*$^{Tg/hUbc\text{-}CreERT2}$ vs *Hnrnpk*$^{Tg/hUbc\text{-}CreERT2}$ MEFs after TMX induction. (B) Left: Representative images of HPG assay confocal microscopy in *Hnrnpk*$^{Tg/hUbc\text{-}CreERT2}$ vs *Hnrnpk*$^{Tg/hUbc\text{-}CreERT2}$ MEFs after

TMX induction; Right: Fluorescence intensity values from the HPG assay (dot-plot, >1.000 cells of representative well; p=0.0001, biological replicate analysis: p=0.0001). (C) Electron micrographs of control MEF (*Hnrnpk*$^{\text{Tg/hUbc-CreERT2}}$) and *Hnrnpk*$^{\text{Tg/hUbc-CreERT2}}$ after TMX induction. (D) Left: Representative images of EU assay confocal microscopy in control MEF (*Hnrnpk*$^{\text{Tg/hUbc-CreERT2}}$) and *Hnrnpk*$^{\text{Tg/hUbc-CreERT2}}$ after TMX induction at 1h, 4h and 24h; Right: Fluorescence intensity values from the EU assay (dot-plot, >1.000 cells of representative well; p=0.0001 biological replicates analysis Control vs 1h: p=0.002; Control vs 4h: p=0.0001; Control vs 24h: p=0.0001). (E) Representative confocal microscopy images of NCL (left panel), FBL (right panel), HNRNPK, and DAPI staining in *Hnrnpk*$^{\text{Tg/hUbc-CreERT2}}$ vs *Hnrnpk*$^{\text{Tg/hUbc-CreERT2}}$ MEFs after TMX induction. Dot-plot analysis of: HNRNPK expression: Alexa Fluor 488 intensity (HNRNPK, dot-plot cell replicates >1.000; biological replicate analysis: p=0.010); NCL relocalization: nucleoplasm Alexa Fluor 647 intensity (NCL, dot-plot cell replicates >1.000; biological replicate analysis: p=0.029) and FBL reload: Alexa Fluor 647 spot total area (FBL, dot-plot cell replicates >1.000; biological replicate analysis: p=0.004). Scale bar: 25µm. (F) *Hnrnpk*$^{\text{Tg/hUbc-CreERT2}}$ vs *Hnrnpk*$^{\text{Tg/hUbc-CreERT2}}$ MEFs after TMX induction qRT–PCR results dot-plot showing pre-rRNA 45S and mature rRNA transcripts 18S, 28S and 5.8S (45S: p=0.0022; 5.8S p=0.0216; 18S: p=NS: 28S: p=NS). Note: Tamoxifen control wild-type cells were additionally used in all experiments, with no significant differences observed (WT + TMX vs *Hnrnpk*$^{\text{Tg/hUbc-CreERT2}}$ + TMX HPG, HNRNPK, NCL, FBL p> 0.05 (NS); WT vs WT+TMX HPG, HNRNPK, NCL, FBL p> 0.05 (NS).

Aguilar-Garrido et al. Supplementary Material. Figure 4

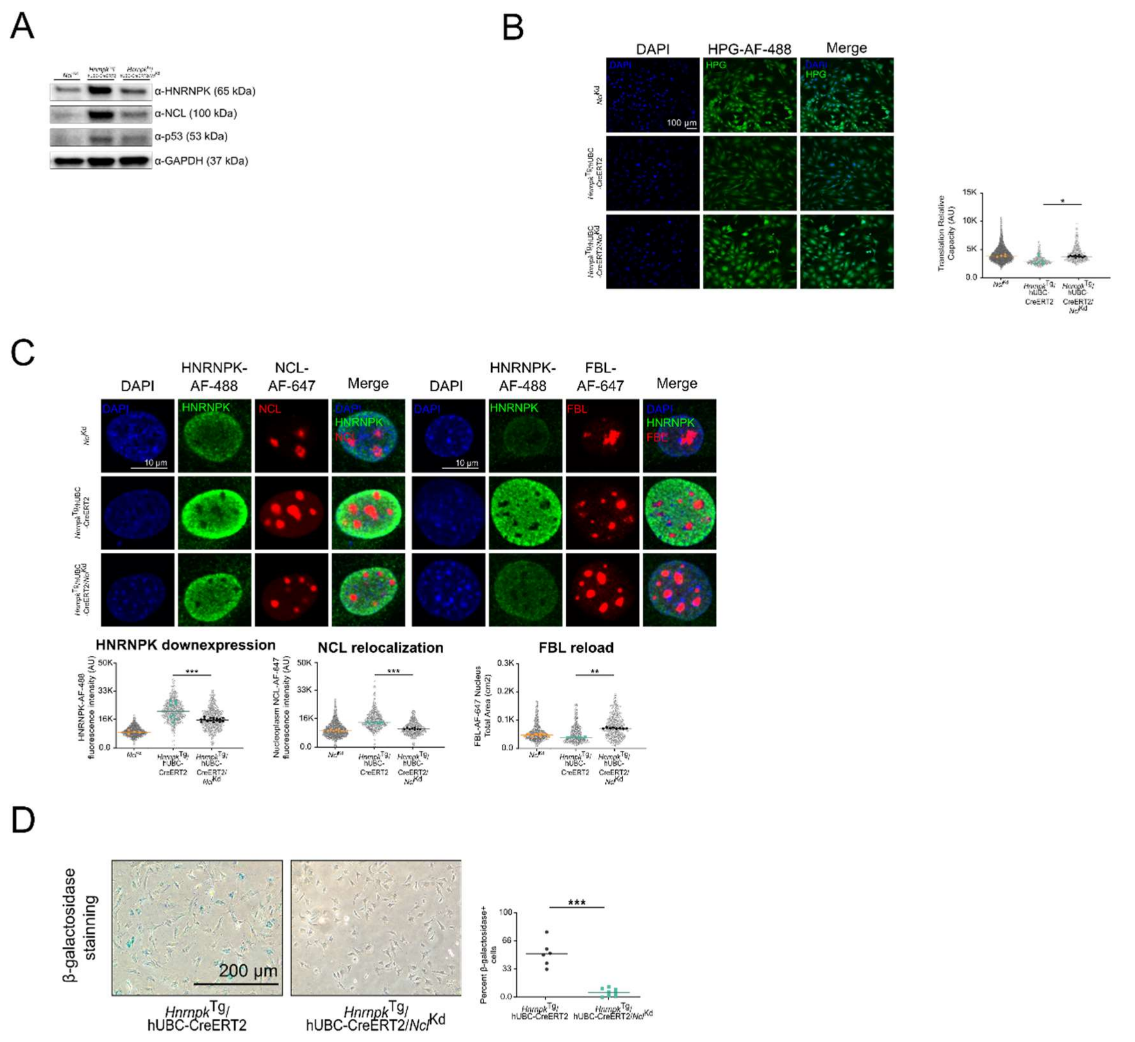

Fig. S4.

(A) Western blot membrane showing HNRNPK, p53 and NCL in *Ncl*^{Kd}, *Hnrnpk*$^{\mathrm{Tg/hUbc\text{-}CreERT2}}$ and *Hnrnpk*$^{\mathrm{Tg/hUbc\text{-}CreERT2}}$/*Ncl*^{Kd} MEFs after TMX induction. (B) Left: Representative images of HPG assay confocal microscopy in *Ncl*^{Kd}, *Hnrnpk*$^{\mathrm{Tg/hUbc\text{-}CreERT2}}$ vs *Hnrnpk*$^{\mathrm{Tg/hUbc\text{-}CreERT2}}$/*Ncl*^{Kd} MEFs after TMX induction; Right: Fluorescence intensity values from the HPG assay (dot-plot, >1.000 cells of representative well biological replicate analysis: p=0.017). (C). Representative confocal microscopy images of NCL (left panel), FBL (right panel), HNRNPK, and DAPI staining in *Ncl*^{Kd}, *Hnrnpk*$^{\mathrm{Tg/hUbc\text{-}CreERT2}}$ and *Hnrnpk*$^{\mathrm{Tg/hUbc\text{-}CreERT2}}$/*Ncl*^{Kd} MEFs after TMX induction. Dot-plot analysis of:

HNRNPK expression: Alexa Fluor 488 intensity (HNRNPK, dot-plot cell replicates >1.000; biological replicate analysis: p=0.0005); NCL relocalization: nucleoplasm Alexa Fluor 647 intensity (NCL, dot-plot cell replicates >1.000; biological replicate analysis: p=0.0007) and FBL reload: Alexa Fluor 647 spot total area (FBL, dot-plot cell replicates >1.000; biological replicate analysis: p=0.036). Scale bar: 25μm. (D) Left: Bright-field microscope images of SA-β-galactosidase staining in *Hnrnpk*$^{\text{Tg/hUbc-CreERT2}}$ vs *Hnrnpk*$^{\text{Tg/hUbc-CreERT2}}$/*Ncl*Kd MEFs. Right: Dot-plot of positive cells for SA-β-galactosidase staining in *Hnrnpk*$^{\text{Tg/hUbc-CreERT2}}$ (n=6) vs *Hnrnpk*$^{\text{Tg/hUbc-CreERT2}}$/*Ncl*^{Kd} (n=8) (p=0.0007). All graphs are shown as the median (B & C) or mean (D). All two groups' statistical analyses were two-sided Student's t-test. For three groups' statistical analyses were two-way ANOVA and Dunn´s multiple comparisons test. . All experiments comprised at least n=3 biological replicates and/or n=3 technical replicates.

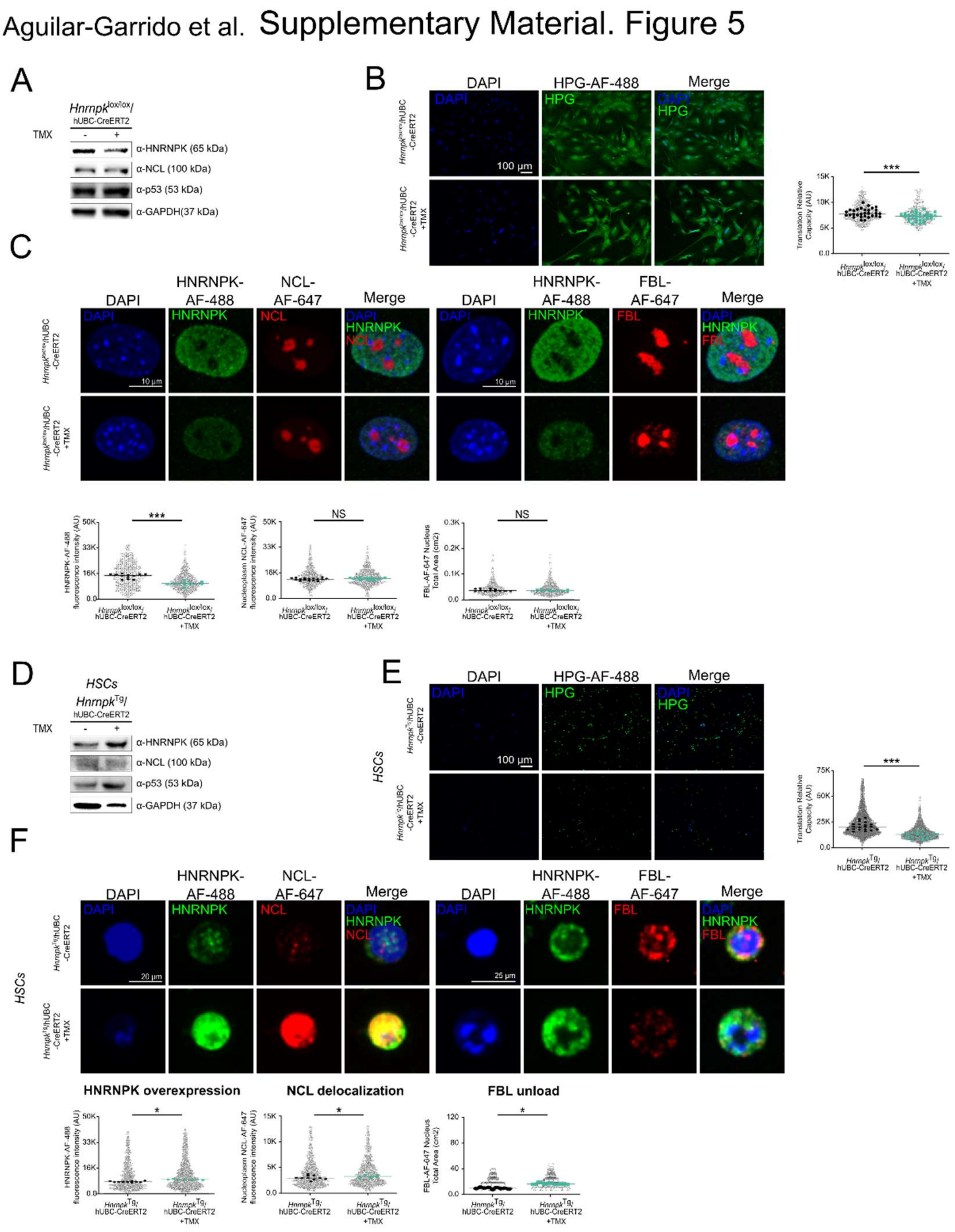

Fig. S5.

(A) Western blot membrane showing HNRNPK, p53 and NCL in *Hnrnpk*$^{lox/lox}$ vs *Hnrnpk*$^{lox/lox}$ MEFs after TMX induction. (B) Left: Representative images of HPG assay confocal microscopy in *Hnrnpk*$^{lox/lox}$ vs *Hnrnpk*$^{lox/lox}$ MEFs after TMX induction; Right: Fluorescence intensity values from the HPG assay (dot-plot, >1.000 cells of representative well, biological replicate analysis: p=0.0001). (C) Representative confocal

microscopy images of NCL (left panel), FBL (right panel), HNRNPK, and DAPI staining in *Hnrnpk*$^{\text{lox/lox}}$ vs *Hnrnpk*$^{\text{lox/lox}}$ MEFs after TMX induction. Dot-plot analysis of: HNRNPK expression: Alexa Fluor 488 intensity (HNRNPK, dot-plot cell replicates >1.000; biological replicate analysis: p=0.0001); NCL relocalization: nucleoplasm Alexa Fluor 647 intensity (NCL, dot-plot cell replicates >1.000; biological replicate analysis: p=NS) and FBL reload: Alexa Fluor 647 spot total area (FBL, dot-plot cell replicates >1.000; biological replicate analysis: p=NS). Scale bar: 25μm. (D) Western blot membrane showing HNRNPK, p53 and NCL in *Hnrnpk*$^{\text{Tg/hUbc-CreERT2}}$ vs *Hnrnpk*$^{\text{Tg/hUbc-CreERT2}}$ HSCs after TMX induction. (E) Left: Representative images of HPG assay confocal microscopy in *Hnrnpk*$^{\text{Tg/hUbc-CreERT2}}$ vs *Hnrnpk*$^{\text{Tg/hUbc-CreERT2}}$ HSCs after TMX induction; Right: Fluorescence intensity values from the HPG assay (dot-plot, >1.000 cells of representative well, biological replicate analysis: p=0.0001). (F) Representative confocal microscopy images of NCL (left panel), FBL (right panel), HNRNPK, and DAPI staining in *Hnrnpk*$^{\text{Tg/hUbc-CreERT2}}$ vs *Hnrnpk*$^{\text{Tg/hUbc-CreERT2}}$ HSCs after TMX induction. Dot-plot analysis of: HNRNPK expression: Alexa Fluor 488 intensity (HNRNPK, dot-plot cell replicates >1.000; biological replicate analysis: p=0.035); NCL relocalization: nucleoplasm Alexa Fluor 647 intensity (NCL, dot-plot cell replicates >1.000; biological replicate analysis: p=0.035) and FBL reload: Alexa Fluor 647 spot total area (FBL, dot-plot cell replicates >1.000; biological replicate analysis: p=0.015). Scale bar: 25μm.

Aguilar-Garrido et al. Supplementary Material. Figure 6

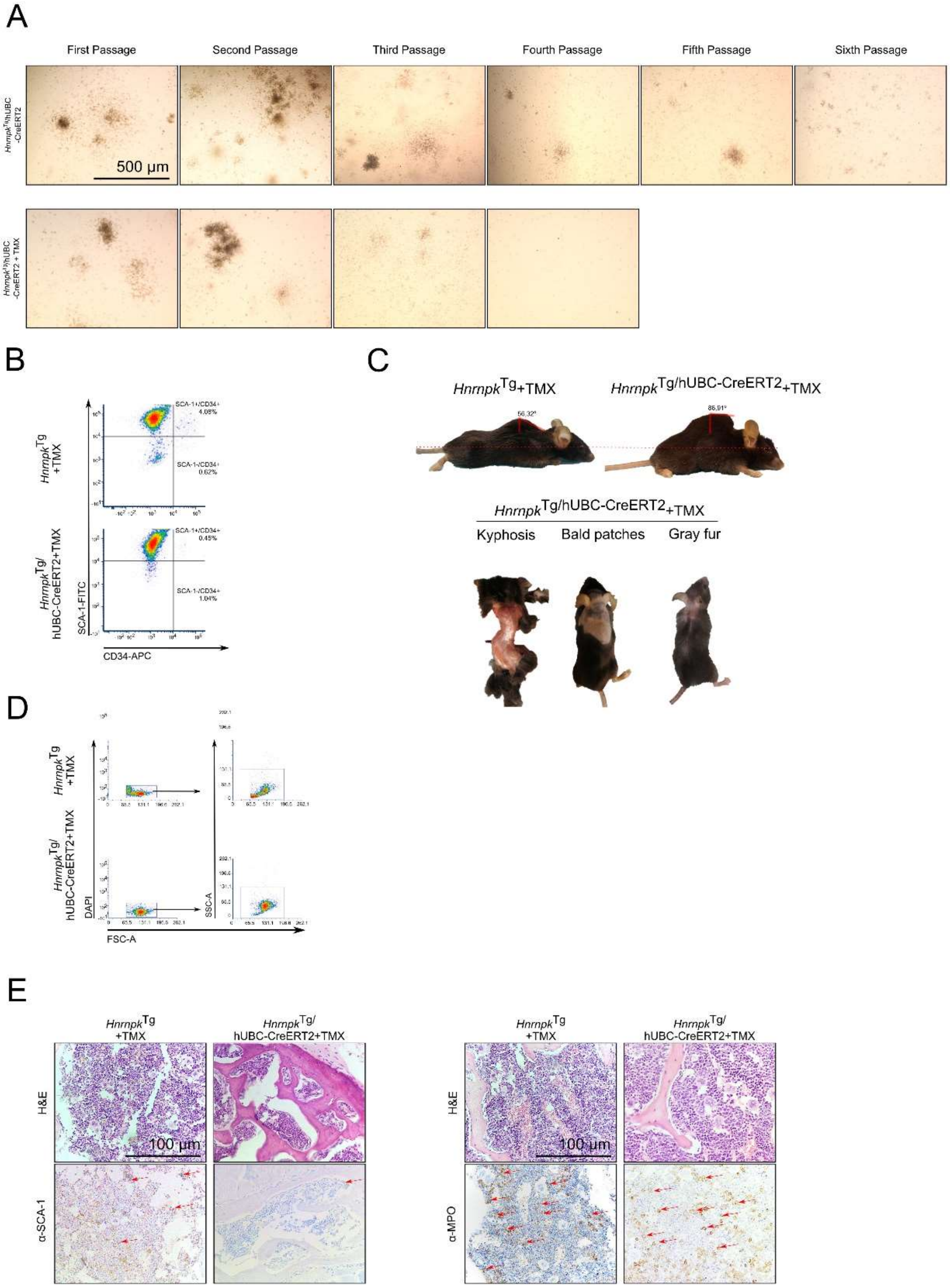

Fig. S6.

(A) Representative microscope images of colony formation unit cultures from 6 passages of the replating assay of *Hnrnpk*$^{Tg/hUbc\text{-}CreERT2}$ HSCs with (n=8) and without (control, n=8) TMX. (B) Representative FCM replating assay (P3), dot plot CD34/SCA-1 analysis of

Hnrnpk$^{Tg/hUbc\text{-}CreERT2}$ CFUs with and without TMX. (C) Representative images of aged-paired TMX-induced *Hnrnpk*^{Tg} and *Hnrnpk*$^{Tg/hUbc\text{-}CreERT2}$ mouse models showing aging phenotypes. Top: kyphosis (increment of backbone curvature: 75% compared to the wild-type). Bottom: Hair issues (bald patches and/or gray fur). (D) Representative sample FCM dot-plot gating strategy from TMX-induced *Hnrnpk*^{Tg} and *Hnrnpk*$^{Tg/hUbc\text{-}CreERT2}$ mouse model total bone marrow cells. (E) HSC and myeloid cells IHC analysis in bone marrow from TMX-induced *Hnrnpk*$^{Tg/hUbc\text{-}CreERT2}$ mice: H&E, SCA-1 and MPO. Scale bar: 100µm. Red arrows: Positive cells. All images are representative of at least n=3 mice and 4 random pathological areas. All experiments comprised at least n>2 biological replicates and/or n>3 technical replicates. Note: *Hnrnpk*^{Tg} + TMX control sample in Figure S6E with Sca-1 staining is the same as that used in Figure 6C (the H&E image is therefore repeated in both figures)..

All tables are included in auxiliary supplementary matherials (excel files)

Table S1.

Gene Set Enrichment Analysis (GSEA) table of significantly regulated pathways from TMTpro results analysis (MsigDB) (FDR q-val<0.25). Normalized enrichment score (NES), positive: overexpressed in Hnrnpk-overexpressing cells; negative: downexpressed in Hnrnpk-overexpressing cells.

Table S2.

Table of significantly regulated proteins from TMTpro results analysis (p-val<0.05). LogFC, positive: overexpressed in Hnrnpk-overexpressing cells; negative: downexpressed in Hnrnpk-overexpressing cells.

Table S3.

Detailed table of patients' clinical data : sex, age at diagnosis, diagnosis, genetic alteration (gene), IHC result of HNRNPK (+/-), % of $CD34^{+}$ cells with IHC $HNRNPK^{+}$.

Table S4.

Gene Set Enrichment Analysis (GSEA) table of significantly regulated pathways from RNAseq results analysis (MsigDB) (FDR q-val<0.25). Normalized enrichment score (NES), positive: overexpressed in Hnrnpk-overexpressing cells; negative: downexpressed in Hnrnpk-overexpressing cells.

Table S5.

Table of significantly regulated genes from RNAseq results analysis (p-val<0.05). LogFC, positive: overexpressed in Hnrnpk-overexpressing cells; negative: downexpressed in Hnrnpk-overexpressing cells.

Key resources table

REAGENT or RESOURCE	SOURCE	IDENTIFIER
**Antibodies**		
Mouse anti-hnRNP K (3C2)	Abcam	Cat# ab39975, RRID: AB_732981
Mouse anti- hnRNP K (D-6)	Santa Cruz Biotechnology	Cat# sc-28380, RRID: AB_627734
Mouse anti-p53 (DO-1)	Santa Cruz Biotechnology	Cat# sc-126, RRID: AB_628082
Rabbit anti-nucleolin	Abcam	Cat# ab22758, RRID: AB_776878
Rabbit anti-fibrillarin (C13C3)	Cell Signaling Technology	Cat# 2639, RRID: AB_2278087
Mouse anti-p16INK4a (1E12E10)	Thermo Fisher Scientific	Cat# MA5-17142, RRID: AB_2538613
Mouse anti-Waf1/Cip1/CDKN1A p21 (F-5)	Santa Cruz Biotechnology	Cat# sc-6246, RRID: AB_628073
Rabbit anti-β-Actin	Cell Signaling Technology	Cat# 4967S, RRID: AB_330288
Rabbit anti-GAPDH (14C10)	Cell Signaling Technology	Cat# 2118S, RRID: AB_561053
Mouse anti-CD34 RTU (QBEND/10)	Leica Biosystems	Cat# PA0212, RRID: AB_10554304
Rat anti-CD34 (RAM34)	Thermo Fisher Scientific	Cat# 14-0341-81, RRID: AB_467209
Rabbit anti-c-Kit (EPR22566-205)	Abcam	Cat# ab231780, RRID: AB_2891166
Rabbit anti-myeloperoxidase	Dako	Cat# A0398, RRID: AB_2335676
Rat anti-Ly-6G/Ly-6C (RB6-8C5)	Invitrogen	Cat# 14-5931-82, RRID: AB_467730
Rat anti-CD11b (M1/70.15)	eBioscience	Cat# 14-0112-82, RRID: AB_467108
Rat anti-CD45R/B220	BD Pharmingen	Cat# 01121A, RRID: AB_394614

Rat anti-p21 (HUGO291)	Abcam	Cat# ab107099, RRID: AB_10891759
Rat anti-β-Galactosidase (3A9A10F8)	CNIO (Spanish National Cancer Research Centre)	N/A
APC anti-mouse CD34	BioLegend	Cat# 128612, RRID: AB_10553896
PE anti-mouse CD117 (c-kit)	BioLegend	Cat# 161503, RRID: AB_2894651
FITC anti-mouse Ly-6A/E (Sca-1)	BioLegend	Cat# 108105, RRID: AB_313342
Brilliant Violet 421™ anti-mouse CD127 (IL-7Rα)	BioLegend	Cat# 135023, RRID: AB_10897948
APC anti-mouse Ly-6G/Ly-6C (Gr-1)	Biolegend	Cat# 108412, RRID: AB_313376
FITC anti-mouse/human CD11b	BioLegend	Cat# 101206, RRID: AB_312788
Brilliant Violet 510™ anti-mouse/human CD45R/B220	BioLegend	Cat# 103248, RRID: AB_2650679
PerCP/Cyanine5.5 anti-mouse TER-119/Erythroid Cells	BioLegend	Cat# 116228, RRID: AB_893638
PE/Cyanine7 anti-mouse CD41	BioLegend	Cat# 133916, RRID: AB_11124102
Donkey anti-Rabbit IgG (H+L) Highly Cross-Adsorbed Secondary Antibody, Alexa Fluor™ Plus 647	Invitrogen	Cat# A32795, RRID: AB_2762835
Donkey anti-Mouse IgG (H+L) Highly Cross-Adsorbed Secondary Antibody, Alexa Fluor™ 488	Invitrogen	Cat# A21202, RRID: AB_141607
Rabbit anti-IgG1 + IgG2a + IgG3 (M204-3)	Abcam	Cat# ab133469, RRID: AB_2910607
Anti-Rat IgG (H+L), mouse adsorbed, made in rabbit	Vector Laboratories	Cat# BA-400, RRID: AB_10015300
Goat Anti-Rabbit Immunoglobulins/HRP	Dako	Cat# P044801-2, RRID: AB_2617138
Goat Anti-Mouse Immunoglobulins/HRP	Dako	Cat# P0447, RRID: AB_2617137

**Bacterial and virus strains**		
lentiMPH v2	Addgene	Cat#89308
lentiSAM v2	Addgene	Cat#75112
**Chemicals, peptides and recombinant proteins**		
4-hydroxytamoxifen diet	Teklad	Cat# 130856
(Z)-4-Hydroxytamoxifen	Sigma-Aldrich	Cat# H7904
Recombinant Murine TPO	PeproTech	Cat# 315-14
Recombinant Murine SCF	PeproTech	Cat# 250-03
Cell Proliferation Reagent WST-1	Sigma-Aldrich	Cat# 11644807001
MethoCult^{TM} methylcellulose medium	STEMCELL Technologies	Cat# GF M3434
DAPI	Invitrogen	Cat# D1306
Actinomycin D	Merck	Cat# A1410550-76-0
IMMAGINA Lysis Buffer	Immagina Biotechnology	Cat# RL001-1
RIPA Lysis Buffer	Millipore	Cat# 20-188
cOmplete^{TM}, Mini Protease Inhibitor Cocktail	Roche	Cat# 11836153001
PhosSTOP^{TM}	Roche	Cat# 4906845001
4x Laemmli Sample Buffer	Bio-Rad	Cat# 1610747
SuperSignal^{TM} West Femto Maximum Sensitivity Substrate	Thermo Fisher Scientific	Cat# 34095
RNeasy Plus Mini Kit	QIAGEN	Cat# 74134
iScript^{TM} cDNA Synthesis Kit	Bio-Rad	Cat# 1708891
SYBR qPCR Master Mix	Promega	Cat# 4367659
Epoxi Dynabeads^{TM} M-270	Invitrogen	Cat# 14301
DynaMag^{TM}-2 Magnet	Invitrogen	Cat# 12321D
Trypsin-EDTA (0.05%), phenol red	Gibco	Cat# 25300096
Blasticidin S HCl	AG Scientific	Cat# B1247
Hygromycin B	Roche	Cat# 10843555001
StemSpan^{TM} SFEM	STEMCELL Technologies	Cat# 09600

Dulbecco′s Modified Eagle′s Medium (DMEM)- high glucose	Sigma-Aldrich	Cat# D5796
Fetal Bovine Serum	Sigma-Aldrich	Cat# F7524
Penicillin/Streptomycin	Solmeglas	Cat# SOPENSRP
Polybrene®	Santa Cruz Technologies	Cat# sc-134220
MagReSyn® Hydroxyl	Resyn Biosciences	Cat# MR- HYX 002
Acclaim™ PepMap™ 100 C18 HPLC Columns	Thermo Scientific	Cat# 164946
**Critical commercial assays**		
Mouse IL-6 DuoSet ELISA	R&D	Cat# DY406
NEBNext® Ultra™ II Directional RNA Library Prep Kit for Illumina®	New England Biolabs	Cat# E7760
Pierce™ BCA Protein Assay Kits	Themo Fisher Scientific	Cat# 23225
TMTpro 18-plex Label Reagents	Thermo Fisher Scientific	Cat# A52045
PhenoPlate™-96	PerkinElmer	Cat# 6055300
Poli-L-lysine solution	Merck	Cat# 8920
Click-iT™ RNA Alexa Fluor™ 488 Imaging Kit	Invitrogen	Cat# C10329
Click-iT™ HPG Alexa Fluor™ 488 Protein Synthesis Assay Kit	Invitrogen	Cat# C10428
Senescence β-Galactosidase Staining Kit	Cell Siganlling Technology	Cat# 9860
Dynabeads™ Co-Immunoprecipitation Kit	Invitrogen	Cat# 14321D
ChromoMap DAB Kit (RUO)	Roche	Cat# 760-159
Bond Polymer Refine Detection	Leica Biosystems	Cat# DS9800
Bond Polymer Refine Red Detection	Leica Biosystems	Cat# D59390
**Deposited data**		
NCBI's Gene Expression Omnibus	This paper	GEO: GSE242038
ProteomeXchange Consortium	This paper	PRIDE: PXD046699
**Experimental models: Cell lines**		
HEK293T	M. Barbacid	RRID: CVCL_0063

Primary *Hnrnpk*$^{Tg\text{-}cre}$ MEFs	This paper	N/A
Primary *Hnrnpk*^{Cre} MEFs	This paper	N/A
Primary *Hnrnpk*$^{Tg\text{-}hUBC\text{-}CreERT2}$ MEFs	This paper	N/A
Primary *Hnrnpk*$^{hUBC\text{-}CreERT2}$ MEFs	This paper	N/A
Primary *Hnrnpk*$^{Tg\text{-}cre}$/*Tp53*$^{\text{lox/wt}}$ MEFs	This paper	N/A
Primary *Hnrnpk*$^{Tg\text{-}CreERT2}$/*Ncl*^{Kd} MEFs	This paper	N/A
Primary *Hnrnpk*^{SAM} MEFs	This paper	N/A
**Experimental models: Organisms/Strains**		
Hnrnpk^{Tg} mice	This paper	N/A
Tg-cre mice	CNIO	N/A
Hnrnpk$^{Tg\text{-}cre}$ mice	This paper	N/A
hUBC-CreERT2 mice	CNIO	N/A
Tp53$^{\text{lox/wt}}$ mice	CNIO	N/A
Hnrnpk$^{Tg\text{-}hUBC\text{-}CreERT2}$ mice	This paper	N/A
Hnrnpk$^{Tg\text{-}hUBC\text{-}CreERT2}$/*Tp53*$^{lox/wt}$ mice	This paper	N/A
**Oligonucleotides**		
Hnrnpk F1 GAAGATATGGAAGAGGAGCAAGCC	Aris et al.(47)	N/A
Hnrnpk R1 CAAGGTAGGGATGATTTTCTTC	Aris et al.(47)	N/A
Cdkn1a F1 TGTCCGTCAGAACCCATGC	Chen et al.(48)	N/A
Cdkn1a R1 AAAGTCGAAGTTCCATCGCTC	Chen et al.(48)	N/A
Cdkn2b F1 AACTCTTTCGGTCGTACCCC	Huda et al.(49)	N/A
Cdkn2b R1 GCGTGCTTGAGCTGAAGCTA	Huda et al.(49)	N/A
Tp53 F1 TGAAACGCCGACCTATCCTTA	Maden et al.(50)	N/A

Tp53 R1 GGCACAAACACGAACCTCAAA	Maden et al.(50)	N/A
Ncl F1 AAAGGCAAAAAGGCTACCACA	Bourbon et al.(51)	N/A
Ncl R1 GGAATGACTTTGGCTGGTGTAA	Bourbon et al.(51)	N/A
Fbl F1 CAAAATTGAGTACAGAGCCTG GA	Aris et al.(47)	N/A
Fbl R1 CGGGCCGACAATATCAGAGA	Aris et al.(47)	N/A
β-actin F1 GGCACCACACCTTCTACAATG	Erson et al.(52)	N/A
β-actin R1 GTGGTGGTGAAGCTGTAGCC	Erson et al.(52)	N/A
Gapdh F1 TCACCACCATGGAGAAGGC	Li et al.(53)	N/A
Gapdh R1 GCTAAGCAGTTGGTGGTGCA	Li et al.(53)	N/A
45S F1 GGCTGGGGTTGGAAAGTTTC	Sirozh et al.(30)	N/A
45S R1 CAAGGGCATTCTGAGCATCC	Sirozh et al.(30)	N/A
18S F1 CTGGATACCGCAGCTAGGAA	Sirozh et al.(30)	N/A
18S R1 GAATTTCACCTCTAGCGGCG	Sirozh et al.(30)	N/A
5.8S F1 GTCGATGAAGAACGCAGCTA	Sirozh et al.(30)	N/A
5.8S R1 AACCGACGCTCAGACAGG	Sirozh et al.(30)	N/A
28S F1 CGGCGGGAGTAACTATGACT	Sirozh et al.(30)	N/A
28S R1 GCTGTGGTTTCGCTGGATAG	Sirozh et al.(30)	N/A

Rplp0 F1 CCCTGAAGTGCTCGACATCA	Gallardo et al.(17)	N/A
Rplp0 R1 TGCGGACACCCTCCAGAA	Gallardo et al.(17)	N/A
Rpl14 F1 GGGTGGCCTACATTTCCTTCG	Blackshaw et al.(54)	N/A
Rpl14 R1 CTTGGCCCATCTTGTGGCT	Blackshaw et al.(54)	N/A
Rpl22 F1 AGCAGGTTTTGAAGTTCACCC	Fujita et al.(55)	N/A
Rpl22 R1 CAGCTTTCCCATTCACCTTGA	Fujita et al.(55)	N/A
Rpl28 F1 GTACAGCACGGAGCCAAATAA	Burke et al.(56)	N/A
Rpl28 R1 GTTTTCGCTGACCGGATCTG	Burke et al.(56)	N/A
Rps3 F1 ATGGCGGTGCAGATTTCCAA	Kim et al.(57)	N/A
Rps3 R1 GTAACTCGGACTTCAACTCCAG	Kim et al.(57)	N/A
Rps9 F1 TTGTCGCAAAACCTATGTGACC	Shibata et al.(58)	N/A
Rps9 R1 GCCGCCTTACGGATCTTGG	Shibata et al.(58)	N/A
Rps12 F1 CTCATCCACGATGGCCTAGC	Ayane et al.(59)	N/A
Rps12 R1 ACATGGGCTCATCACAGTTGG	Ayane et al.(59)	N/A
Rps16 F1 CACTGCAAACGGGGAAATGG	Meyuhas et al.(60)	N/A
Rps16 R1 CACCAGCAAATCGCTCCTTG	Meyuhas et al.(60)	N/A
Rps21 F1 GTCCATCCAGATGAACGTGG	Trinidad et al.(61)	N/A
Rps21 R1 CCATCAGCCTTAGCCAATCGG	Trinidad et al.(61)	N/A

sg*Hnrnpk1* CACCGCGCTGCTCACGTGTGCC GGG	This paper	N/A
sg*Hnrnpk2* CACCGCCGAGGGAGTTTGGCG CGAT	This paper	N/A
sgNon-Targeting (sgNT) CACCGCCGAGGGAGTTTGGCG CGAT	This paper	N/A
Hnrnpk F_30F12 CCAGATACAGAACGCACAGT	This paper	N/A
pCALL:R_30F13 AAGGGGCTTCATGATGTCC	This paper	N/A
pCALL:S_30F14 Fam-CTCGAGGTGGCTGCGATC-Zen-IBFQ	This paper	N/A
*p53*Flox-F_1F10 GGAATACTTCAAGAGACGGAG A	This paper	N/A
*p53*Flox-R_1F11 AGCCAGGACTACACAGAGAA	This paper	N/A
*p53*Flox-wt_1F13 Hex-AAATTATGATTCGAACAGAAT AAAGGATT-Zen-IBFQ	This paper	N/A
*p53*Flox-lox_1F12 Fam-CTGCAGATAACTTCGTATAGCA TACAT Zen-IBFQ	This paper	N/A
**Recombinant DNA**		
pCALL2 vector	Lobe et al.(62)	N/A
pCALL2-Hnrnpk	This paper	N/A
**Software and algorithms**		
Adobe Photoshop 24.0.0	Adobe Systems	RRID: SCR_014199, https://www.adobe.com/products/photoshop.html

FCS Express^{TM} 7 Software	De Novo Software	RRID: SCR_016431, https://denovosoftware.com/?gclid=EAIaIQobChMI36rn3-Dd3AIV2ud3Ch27lw2oEAAYASAAEgLbRvD_BwE
FastQC 0.11.0	Andrew S.	RRID: SCR_014583, http://www.bioinformatics.babraham.ac.uk/projects/fastqc/
TopHat2	Trapnel et al.(36)	RRID: SCR_013035, http://ccb.jhu.edu/software/tophat/index.shtml
Bowtie2	Langmead et al.(37)	RRID: SCR_016368, https://bowtie-bio.sourceforge.net/bowtie2/index.shtml
SAMTOOLS	Li et al.(63)	RRID: SCR_002105, https://www.htslib.org/
Gencode vM29	Frankish et al.(64)	RRID: SCR_014966, https://www.gencodegenes.org/
HTSeq	Anders et al.(39)	RRID: SCR_005514, https://htseq.readthedocs.io/en/release_0.9.1/
DESeq2	Love et al.(40)	RRID: SCR_004463, https://bioconductor.org/packages/release/bioc/html/DESeq2.html
MaxQuant 2.1.4.0	Sinitcyn et al.(65)	RRID: SCR_014485, https://www.maxquant.org/archive/maxquant
Prostar package 1.22.3	Wieczorek et al.(42)	RRID: XXX, https://bioconductor.org/packages/release/bioc/html/Prostar.html

GSEA 4.3.2	Subramanian et al.(21)	RRID: SCR_003199, http://www.broadinstitute.org/gsea/
Harmony 5.1/Acapella 2.6	Perkin Elmer	RRID: XXX, SCR_023543, https://www.perkinelmer.com/product/harmony-5-1-office-hh17000012
Gatan Microscopy Suite	Gatan	RRID: SCR_014492, https://www.gatan.com/products/tem-analysis/gatan-microscopy-suite-software
Prism 7.0	GraphPad	RRID: SCR_002798, https://www.graphpad.com/scientific-software/prism/
CRISPR-ERA design tool	Stanford University	RRID: SCR_018710, http://crispr-era.stanford.edu/index.jsp
Perseus 1.6.7.0	MaxQuant	RRID: SCR_015753, https://maxquant.net/perseus/
